# Supplementary material for: Inferring the molecular and phenotypic impact of amino acid variants with MutPred2
Source: Nat Commun. 2020 Nov 20;11:5918. doi: 10.1038/s41467-020-19669-x (PMC7680112; doi:10.1038/s41467-020-19669-x)
Supplement: Supplementary file 1 — Supplementary Information [file 41467_2020_19669_MOESM1_ESM.pdf]

Supplementary Information

Inferring the molecular and phenotypic impact of amino acid variants with MutPred2

Vikas Pejaver, Jorge Urresti, Jose Lugo-Martinez, Kymberleigh A. Pagel, Guan Ning Lin, Hyun-Jun Nam, Matthew Mort, David N. Cooper, Jonathan Sebat, Lilia M. Iakoucheva, Sean D. Mooney, Predrag Radivojac

Table of Contents

1 Supplementary Methods 1
1.1 Development of property predictors 1
1.1.1 Data collection and preprocessing 1
1.1.2 Common feature set 2
1.1.3 Model selection 2
1.1.4 Training 3
1.2 MutPred2 predictor development 7
1.2.1 Data collection and preprocessing 7
1.2.2 Feature extraction 7
1.2.3 Model-specific features 9
1.2.4 Evaluation 10
1.2.5 Evaluation on a prospectively collected independent data set 11
1.2.6 MutPred2.1 11
1.3 Positive-unlabeled predictors for ranking mechanisms 12
1.3.1 Data sets 12
1.3.2 Training and evaluation 12
1.3.3 Estimation of class prior probabilities 13
1.3.4 Transformation to posterior distribution 14
2 Supplementary Notes 15
2.1.1 Comparison with MutPred 15
2.1.2 Prospective evaluation and updated training sets 15
2.1.3 Clustering of disease categories based on affected properties 16
3 Supplementary Tables 17
4 Supplementary Figures 32
5 Supplementary Data 41
Supplementary References 42

1 Supplementary Methods

1.1 Development of property predictors

1.1.1 Data collection and preprocessing

Training data sets for various property predictors developed in this work were obtained from public databases and the literature. In order to construct a predictor of relative solvent accessibility (RSA), we used the data set constructed by Naderi-Manesh et al.[1] For the simultaneous prediction of signal peptide and transmembrane topology, we downloaded the data set that was used to train the Phobius predictor.[2] MARCOIL's training data was used to develop a predictor of coiled-coil regions.[3] The catalytic residue, DNA-binding, metal-binding and protein-protein interaction (PPI) data sets were obtained from the study by Xin.[4] For RNA-binding sites, the RB199 data set was downloaded from the Protein-RNA Interface Database.[5] The data set collected by Agius et al. served as the source for PPI hotspot information.[6] The Molecular Recognition Feature (MoRF) data set constructed by Disfani et al. was utilized for the development of a MoRF predictor.[7] Finally, allosteric sites were obtained from the AlloSteric Database (January 2014).[8]

In cases of properties where the data were obtained from three-dimensional structures (Supplementary Table 2), we mapped the positions of residues of interest in the crystal structures' records back to the full-length sequences in the SEQRES field and used these sequences for training. In other cases, mapping to sequences were either readily available, or were mapped to the sequences from UniProt.[9] In the following sections, we refer to structural or functional sites of interest as positive sites (class label: 1), and other residues (either a remainder or a subset thereof) as negative sites (class label: 0). In the case of functional properties, we removed redundant examples based on sequence identity in the local neighborhood to achieve better generalization. Each positive and negative site was associated with a 25-residue fragment centered at the residue of interest (for the sites near termini, the fragments were asymmetric). We removed all residues associated with fragments that were more than 40% identical to other fragments in the data set. In cases where a fragment containing a negative site was 40% identical to a fragment containing a positive site, the one with the negative class label was removed because its class designation was less reliable. No such neighborhood-based redundancy removal was performed for the structural property prediction tasks. This was because these data sets were already non-redundant at the level of entire sequence and large enough for us not to be concerned with overfitting. Other property-specific modifications to the protocol (if any) are discussed in Section 1.1.4. The final data set sizes are shown in Supplementary Tables 3-4.

1.1.2 Common feature set

For all property predictors developed for MutPred2, we extracted and encoded a common set of features. These can be broadly divided into three groups: (1) sequence-based features; (2) features based on physicochemical and other predicted properties; and (3) conservation-based features.

Sequence-based features included relative amino acid frequencies, beta entropies[10] ($\beta \in \{1, 1.25, 1.50, 1.75\}$), proportions of aromatic residues (F, Y and W), charge-hydrophobicity ratios[11] and net and total charges calculated using concentric windows centered at the residues of interest. Net and total charges were calculated by taking the difference and sum of the counts of positively charged (K and R) and negatively charged residues (D and E) residues within these windows. For this set of features, we used windows of sizes $w \in \{3, 7, 11, 21\}$. This resulted in a set of 112 features for each training example. We then encoded the presence or absence of the 20 possible amino acids within three positions N-terminal and C-terminal to the residue of interest using a binary representation (presence = 1; absence = 0). This added another 120 features to the feature set.

The second set of features included physicochemical properties such as flexibility,[12] hydrophobic moment (at rotation angles 100, 120 and 160 degrees),[13] amino acid volume,[14] hydrophobicity[15] and predicted structural properties such as intrinsic disorder (VL2[16] and VSL2B[17]), B-factor[18] and secondary structure (inhouse predictor). In the case of the functional property predictors, relative solvent accessibility predictions from the classifier developed in this work were also included (Section 1.1.4). These features were calculated or predicted for each residue within windows of sizes $w \in \{1, 7, 11, 21\}$ and then encoded by taking the mean, the standard deviation, and maximum values over each window. These accounted for 180 and 192 features in the common feature set for the structural and functional property predictors, respectively.

The third set of features was intended to represent evolutionary constraints around sites of interest. To obtain these, position-specific scoring matrices (PSSMs) were first constructed for full-length protein sequences by running PSI-BLAST (v.2.2.18; E-value threshold: 0.0001; number of passes: 3) against the NCBI non-redundant database (June 2013).[19] Then, features were extracted by averaging values within every column of the PSSM, around the residue of interest using window sizes $w \in \{1, 3, 11, 21\}$. Thus, 168 features were added to the common feature set.

1.1.3 Model selection

For the majority of property predictors, we used 10-fold cross-validation to evaluate the performance of different learning algorithms and parameters (see Section 1.1.4 for exceptions). The folds were defined such that all examples from a protein were either entirely in the training set or the test set. To measure performance, areas under the ROC curve (AUCs) were used as the primary performance measure.

For each property, we compared the performance of three different learning algorithms: a bagged ensemble of logistic regression models, a bagged ensem-

ble of neural networks and a random forest; i.e., ensembles of linear classifiers, ensembles of universal approximators, and a learning algorithm shown to be robust in practice for ranking purposes. First, we started with fixed parameter sets for all algorithms and selected the best-performing algorithm based on the above cross-validation procedure. Then, we further optimized this algorithm by using an ad hoc approach where the effect of changing specific training parameters was measured while keeping the others constant. Although more sophisticated optimization approaches exist, given our common feature set and varying sizes of training data sets, such approaches are likely to lead to overly complex models with a poor tendency to generalize. Furthermore, we note that the performances of the different learning algorithms were similar, suggestive of stable solutions that were unlikely to change through additional parameter optimization.

During the model selection process, certain settings were used as defaults for each algorithm. Logistic regression models were trained in an iterative manner using the maximum conditional likelihood approach. All neural networks were feed-forward networks with a single hidden layer and the hyperbolic tangent sigmoid (tansig) function was used as the transfer function in the hidden and output layers. Neural networks were trained using resilient propagation[20] and stoppage criteria were obtained based on a validation subset extracted from the training set in each fold. In the case of random forests, regression trees were trained in order to obtain a smoother prediction score distribution. While all trees were allowed to grow fully without any pruning, the minimum number of examples per leaf node was allowed to be three to alleviate overfitting. The number of features randomly selected for each split (the *m* parameter) was set to be the square root of the number of features. Furthermore, categorical features were treated as such. In the case of logistic regression and neural networks, an ensemble of size 30 was chosen and for the random forests, 100 trees were trained.

We also considered other important issues related to the practical aspects of training. These were adhered to during both evaluation and final training. First, to address class imbalance in all of the above data sets, we trained each member of an ensemble with a balanced set, such that the same number of positives and negatives were presented to any learner; i.e., the majority class was down-sampled after the original data set was randomly sampled with replacement. Second, to avoid numerical stability issues, such as near-singular Hessian matrix, and to prepare the data for logistic regression and neural networks, we z-score normalized original data sets and performed principal component analysis (PCA) on these data to eliminate co-linear and nearly co-linear features. Additionally, we performed feature selection using a two-sample t-test to remove constant and irrelevant features. Finally, to avoid information leak during evaluation, all of these procedures were run on the training partition only and the resulting outputs (e.g., normalization and transform matrices for PCA) were then applied to the test data.

1.1.4 Training

In this section, we discuss the details of the final model for each property predictor. We further discuss any deviations from the feature extraction, model selec-

tion, model optimization and evaluation protocols. A general summary of the different property predictors developed as part of this work is shown in the Supplementary Tables 3-4.

Coiled-coil regions. The training protocol for the coiled-coil region predictor was different from the protocols mentioned in the previous sections, as we aimed simply to re-implement MARCOIL inside MutPred2. To this end, we followed the data preprocessing, training and evaluation procedures described by Delorenzi and Speed.[3] Briefly, we first removed homologous sequences from the training set by filtering out sequences at 70% identity (note: this cutoff was more stringent than that in the original protocol; however, a comparable number of sequences were retained). The resulting data set comprised 286 protein sequences with annotated coiled-coil regions, 73 of which were of human origin. Next, we obtained background and emission probabilities from the files provided with the COILS program.[21] Then, we implemented the hidden Markov model (HMM) designed by Delorenzi and Speed and evaluated it in a leave-one-out cross-validation procedure. As emphasized in the original protocol, training in each fold started with the same set of transition probabilities. Then, the Baum-Welch algorithm was utilized to derive updated transition and emission probabilities, which were then used to make predictions on the test set.[22] After evaluation, a single HMM was trained to predict both coiled-coil regions and their internal heptad structures from protein sequence. However, for MutPred2, a simple binary classification mode (coiled-coil or not) was chosen. This was achieved by taking the complement of the probability of the background (non-coiled coil) state for every residue; i.e., the sum of the probabilities of all the possible heptad states.

Relative solvent accessibility. Although RSAs of individual residues are typically represented as real-valued quantities, we found that performance was poor when using regression or multiclass classification. Therefore, we adopted the commonly chosen binary classification approach; i.e., predicting whether a residue is buried or exposed. To assign residues in our training data to one of these classes, we first obtained absolute solvent accessibility values from the structures in our data set using the DSSP program.[23] Then, for every residue, we derived its RSA by dividing its absolute accessibility with its maximal accessibility, obtained from previous works.[24, 25] Finally, as outlined by Rost and Sander, we used an RSA cutoff value of 0.16 to define whether a residue was buried or exposed.

We noted that secondary structure features were not included in the final feature set for this predictor. As mentioned above, our initial attempts to build models were aimed at predicting either the actual RSA value or one of multiple class labels. Bagged neural networks were ideally suited for these tasks. For reasons of convenience, we retained this approach even for the binary classification problem and did not use the model selection protocol discussed in Section 1.1.3. However, in a post hoc analysis, we compared this approach to that of using random forests and found that the results were similar. Before training the final model, feature selection at a significance threshold of 1×10^{-8} was performed,

followed by normalization and PCA (retained variance: 99%). The final model was an ensemble of 30 bagged neural networks with 16 hidden neurons in each of them. Training was stopped either after 1000 iterations or if the maximum number of validation checks was 100.

Signal peptide/transmembrane segment topology. The prediction of transmembrane (TM) helical segments is often confounded by the presence of helical regions in signal peptides in many such proteins. Therefore, a common approach is to train models that not only predict TM segment topology but also distinguish between TM and signal regions. Since this was a special case, we treated it as a multiclass classification problem where a residue could be assigned to one of eight classes: N-terminal region of the signal peptide, signal helix, C-terminal region of the signal peptide, signal peptide cleavage site, cytoplasmic loop, TM helix, non-cytoplasmic loop or none of these; i.e., when the given protein is not a TM protein.

While the features used in training were the same as those discussed in Section 1.1.2, to model conservation over larger distances, window sizes $w \in \{1, 7, 11, 21\}$ were used for PSSM-based features. Before training, normalization and PCA (retained variance: 99%) were performed to remove highly correlated features. We note that no feature selection was performed before this and all features were included. The final model was an ensemble of 30 bagged neural networks, each with 64 hidden neurons. Unlike the standard protocol (Section 1.1.3), networks were trained on stratified samples of the training data set; i.e., for a given class, the same fraction of examples, as that in the original training data set was presented to each network. Training was stopped either after 1000 iterations or if the maximum number of validation checks was 200.

Allosteric site. For this task, no feature selection or normalization was performed. The final model chosen was a random forest with 100 trees with the *m* parameter set to three times the square root of the size of the feature set. In contrast to the default model (Section 1.1.3), categorical features were treated here as continuous numerical features that were automatically thresholded by the learning algorithm during training.

Catalytic residue. The best model in this case was an ensemble of 30 logistic regression models. No feature selection was carried out before training but the feature matrix was normalized and PCA was applied (retained variance: 99%). Training was stopped either after 1000 iterations or if there was very little difference in weights (1×10^{-10}) between iterations.

DNA-binding residue. Before training, feature selection at a significance threshold of 0.01 was performed, followed by normalization and PCA (retained variance: 99%). Although we started with a bagged logistic regression ensemble consisting of 30 models, we eventually trained a single logistic regression model, as its performance was similar to that of the ensemble. Training was stopped either after 1000 iterations or if there was very little difference in weights (1×10^{-10}) between iterations.

Metal-binding residue. Training data sets for 11 different types of metal-binding site were obtained and the aforementioned protocols in Sections 1.1.1, 1.1.2 and 1.1.3 were applied. Depending on the metal, either neural network ensembles or random forests emerged as the best-performing models. For each model type, a general set of parameters was derived over all metals and was used to train the final models. Although this simplification could potentially result in sub-optimal parameters for specific metal types, metal-specific tuning of parameters yielded only very small improvements in performance.

The metals for which neural network ensembles were trained were cadmium, copper, iron and manganese. Before training, feature selection at a significance threshold of 1 x 10^{-4} was performed, followed by normalization and PCA (retained variance: 99%). The ensemble size in these cases was 30 and each network had four hidden neurons. Training was stopped either after 1000 iterations or if the maximum number of validation checks was 100. The metals for which random forest models were trained were calcium, cobalt, magnesium, nickel, potassium, sodium and zinc. In this case, no feature selection or normalization was performed. The final model contained 100 trees with the *m* parameter set to the square root of the size of the feature set. We note that, although metal ion coordination occurs at specific residues, metal-binding sites in the training data were defined in terms of their proximity to metal ions in three-dimensional space. Therefore, training and, subsequently, prediction were performed on every residue of a sequence without regard to amino acid type.

MoRF. For this task, the best model was a random forest with the default parameters (Section 1.1.3) with no feature selection or normalization; i.e., 100 trees with the *m* parameter set to the square root of the size of the feature set.

PPI residue. PPI residues in the training data were defined in terms of their proximity to atoms from the partner protein in three-dimensional space and that this may not necessarily be indicative of their roles in binding. Owing to this fact, there remained a possibility that residues adjacent to PPI residues were incorrectly labeled as belonging to the negative class. To address this, all negative sites within three residues or less of a positive site were excluded from training. Before training the final model, feature selection at a significance threshold of 1 x 10^{-12} was performed, followed by normalization and PCA (retained variance: 99%). The final model was an ensemble of 30 bagged neural networks, each with 16 hidden neurons. Training was stopped either after 1000 iterations or if the maximum number of validation checks was 100.

PPI hotspot. The original training data set contained hotspot information in terms of $\Delta\Delta G$ values obtained from alanine scanning experiments. As proposed by the authors of the original data set, we defined hotspots as those residues that resulted in $\Delta\Delta G$ values of at least 2 kcal/mol and the rest as non-hotspot residues.[6] The particularly difficult nature of this prediction problem necessitated the inclusion of two additional features. First, since residues that are hotspots for PPIs are

by default involved in PPIs, we included scores from the aforementioned PPI predictor. Second, since the data were obtained from thermodynamic studies on single-residue alanine mutations, we artificially mutated each residue in the training data set to alanine and obtained scores from a re-implemented version of the MUpro stability predictor.[26]

We note that the use of larger window sizes negatively impacted prediction performance. This may have been because hotspots in the training data were identified through single-residue mutation studies and the inclusion of neighborhood features were confounders for the learner. Therefore, only features encoding the actual site; i.e., window size of 1, were included. Before training, feature selection at a significance threshold of 0.01 was performed, followed by normalization and PCA (retained variance: 99%). The final model was a bagged logistic regression ensemble consisting of 50 models. Training was stopped either after 100 iterations or if there was very little difference in weights (1×10^{-10}) between iterations.

RNA-binding residue. Before training the final model, feature selection at a significance threshold of 0.01 was performed, followed by normalization and PCA (retained variance: 99%). The final model was an ensemble of 30 bagged neural networks with four hidden neurons in each of them. Training was stopped either after 1000 iterations or if the maximum number of validation checks was 500.

1.2 MutPred2 predictor development

1.2.1 Data collection and preprocessing

We created a data set of disease and unlabeled (putatively non-disease) variants by integrating data from different sources: HGMD (June 2013), Swiss-Prot (Release 2012_09 through SwissVar) and dbSNP (build 137). While disease substitutions came from all three sources, non-disease variants were compiled from Swiss-Prot and dbSNP only. We supplemented the non-disease set with additional variants by including residues at positions in human proteins that differed from those in highly similar proteins in other species. Specifically, for every human protein, we first extracted pairwise alignments to other mammalian proteins from a 46-species multiple sequence alignment, obtained from the UCSC Genome Browser.[27] Then, we only considered those alignments where the two sequences were at least 99% identical to each other and identified positions where a residue in the non-human sequence is replaced by a different one in the human sequence. We reasoned that such changes in highly conserved sequences are less likely to be disease-causing because they have been tolerated over a long period of time since the speciation event.

1.2.2 Feature extraction

The MutPred2 feature set comprises the set of features from the original MutPred model with differences in representation and several new additions. In general, the MutPred2 feature set can be divided into six groups: (1) sequence-based fea-

tures, (2) substitution-based features, (3) PSSM-based features, (4) conservation-based features, (5) homolog counts and (6) changes in predicted structural and functional properties.

The sequence-based features correspond to a combination of the first group of features and the physicochemical properties from the second group described in Section 1.1.2. Similarly, the PSSM-based features are the same as the conservation-based features described in Section 1.1.2, with differences only in the window-sizes used; $w \in \{1, 5, 11, 21\}$.

We represented the variants in three different ways. First, we extracted the score corresponding to the wild-type and substituted amino acid from different BLOSUM[28] and PAM[29] substitution matrices. The matrices used were BLOSUM30 to BLOSUM90 (in increasing intervals of 5), BLOSUM62, BLOSUM100 and PAM10 to PAM500 (in increasing intervals of 10). Each score represents the likelihood of an amino acid being substituted by another, independent of its position in the protein. Additionally, to capture position-independent physicochemical effects of a variant, we carried out the same steps for the Grantham matrix.[30] Second, we created a sparse binary vector encoding all 20 x 19 possible combinations of wild-type and substituted residue pairs and set the element corresponding to the substitution as one. Finally, to approximate the likelihood of observing a substitution, we computed transition frequencies for both wild-type and substituted residues, as in SNAP[31] and MutPred.[32]

In order to encode local conservation around a substituted position, we used the AL2CO program to calculate nine different conservation indices for every position in the 46-species alignments obtained from the UCSC Genome Browser.[33] Both, the normalized and unnormalized versions of these nine scores were calculated. Then, features were extracted by averaging index values over windows of sizes $w \in \{1, 5, 11, 21\}$ around the position of the variant. Additionally, the frequencies of the original and substituted residue in the alignment column were included as features (with and without gaps separately). All conservation-based features were extracted from the full alignment and also from sub-alignments consisting of sequences from only primates and only mammals, as in previous work.[34]

It has previously been shown that genes with highly similar paralogs are less likely to harbor disease mutations.[35] To capture this information for a given variant, we first aligned its parent sequence to all remaining human proteins (canonical isoforms only) using the Needleman-Wunsch algorithm[36] and then included the number of homologs at different sequence identity thresholds (50% to 95% in intervals of five). This procedure was extended to mouse proteins as well, resulting in two vectors of counts each of length 10. We refer to the resulting 20 features as homolog counts or homolog count profiles.

We modeled the local effects of the variant on predicted structural and functional properties as follows. We first input the wild-type sequence to the various predictors included in MutPred2 and obtained scores at the substitution site and all residues within ±5 positions of it. We then introduced the variant into the sequence in silico and input the new sequence to all the property predictors. From

these scores, the probabilities of changes in properties, given the substitution were calculated as follows:

$$\begin{aligned}\Pr(\text{loss of property } p \mid s, XiY) &= \Pr(\text{presence of property } p \mid s) \cdot \Pr(\text{absence of property } p \mid s_{XiY}) \\ &= \Pr(P=1 \mid s) \cdot (1 - \Pr(P=1 \mid s_{XiY}))\end{aligned} \quad (1)$$

and

$$\begin{aligned}\Pr(\text{gain of property } p \mid s, XiY) &= \Pr(\text{absence of property } p \mid s) \cdot \Pr(\text{presence of property } p \mid s_{XiY}) \\ &= (1 - \Pr(P=1 \mid s)) \cdot \Pr(P=1 \mid s_{XiY})\end{aligned} \quad (2)$$

In the above equations, when the wild-type residue at the *i*-th position of the protein is *X* and the replacement amino acid is *Y*, the score from the predictor for property *p* when run on the wild-type sequence can be interpreted as $\Pr(P=1 \mid s)$ and the score when run on the substituted sequence can be treated as $\Pr(P=1 \mid s_{XiY})$. In the final feature set, for every property, we chose the position within the 11-residue window that corresponded to the highest impact (loss or gain) and included the wild-type score, score after substitution, the probability of loss and the probability of gain.

In addition to the structural properties derived from predictors developed in this work (Section 1.1), we used or re-implemented existing predictors for intrinsic disorder,[17] secondary structure (in-house), B-factor[18] and stability (model derived from MUpro[26]). We did the same for the functional property predictors and used existing sequence-based predictors for calmodulin-binding[37] and 22 types of post-translational modification (PTM) sites.[38] In the case of stability, no losses or gains were computed and the classification score from the predictor was directly used. We also encoded the impact of substitutions on sequence motifs obtained from the PROSITE[39] and ELM[40] databases. Regular expression matching was used to check if a substitution occurred within a motif and the number of such motifs was counted. A logistic transformation was applied to assign scores between zero and one and, subsequently, losses and gains were calculated as before. In total, 53 properties were considered (Supplementary Table 1).

1.2.3 Model-specific features

We refer to the model trained on the above feature set as the main MutPred2 model. It is important to note that homolog counts described in the previous section are protein-level features and are not informative when attempting to distinguish between disease-causing and neutral variants in the same protein. Furthermore, in practical settings, the computation of global sequence alignments for new sequences is expensive. Nevertheless, these features approximately account for the prior probability of a variant to be involved in disease and can, thus, rescale prediction scores for individual variants. Therefore, the need for the inclusion or exclusion of homolog counts as features may largely depend on the use case. To allow for such flexibility, we trained an additional model without these features and refer to it as the model without homolog counts.

It is well-established that positional (evolutionary) conservation is highly discriminative in the task of classifying amino acid variants. However, in some cases, owing to the lack of homologs or the unavailability of pre-computed alignments for some proteins (e.g., alternate isoforms), these features simply cannot be calculated. To ensure that every input variant has a prediction, we trained a regression model for the prediction of conservation features from PSI-BLAST PSSMs. Specifically, two multi-output neural network ensembles were trained: one for unnormalized conservation scores and another for residue column frequencies. The features for these predictors were obtained as follows. First, for each human protein with a UCSC alignment, 15 positions were randomly chosen. Then, for each position, its corresponding vector in the PSSM was extracted. In addition, the position itself was encoded as absolute and relative (to the length of the protein) values, along with the amino acid at the position (encoded as a 20-element binary vector). The final training data set consisted of 444,660 such 64-element feature vectors. Two prediction target matrices were constructed for this training set. First, for each position, six types of frequencies were obtained from the UCSC alignments, with and without gaps from three types of alignments (as discussed in Section 1.2.2). Second, as discussed previously, nine conservation scores (unnormalized only) at the corresponding positions were obtained using AL2CO on the three types of alignments.

The prediction of these 120 frequency values and 27 conservation scores was treated as two separate multi-target regression tasks and two corresponding neural network ensembles were trained. Except for the number of outputs, the training protocols for both ensembles were the same. We note that this protocol was optimized in a manner similar to that outlined in Section 1.1.3. Each ensemble consisted of 30 feed-forward neural networks trained on different bootstrap samples of the training data set. Each network had a single hidden layer (64 neurons) with the tansig transfer function and an output layer with a linear transfer function. The networks were trained using resilient propagation with the stoppage criteria set to 1000 iterations or 500 validation checks. Although no PCA or feature selection was performed before training, both the feature and target matrices were z-score normalized.

We note that a separate model was not trained for the normalized versions of the above conservation scores. Instead, the predicted scores were simply z-score normalized to complete the conservation-based feature set. In this manner, prediction models could be used to compensate for the lack of the aforementioned conservation-based features, albeit with some error. Therefore, in addition to the main model and the model with homolog counts, two more models were trained: predicted conservation, with homolog counts and predicted conservation, without homolog counts. Again, depending on the use case, any of these models can be invoked in MutPred2.

1.2.4 Evaluation

To ensure unbiased performance estimates, all models were evaluated using a special 10-fold cross-validation procedure similar to that in previous work.[41] Briefly, all sequences in the training set were first clustered together using CD-HIT

such that no two proteins from different clusters were more than 50% identical to each other.[42] Then, during cross-validation, it was ensured that all variants from a cluster either belonged entirely to the training set or the test set. ROC curves and related performance measures were derived in the same manner as described in Section 1.1.3.

1.2.5 Evaluation on a prospectively collected independent data set

A new independent test set was compiled from mutations deposited in HGMD (Professional version 2017.1) and gnomAD (VCF files from, release 2.1). To map variants represented as chromosomal co-ordinates in gnomAD to amino acid substitutions, Ensembl IDs for the canonical isoform provided in the VCF files were used. For practical purposes, instead of using the entire gnomAD resource, 50 substitutions from each protein sequence were randomly sampled (if a sequence harbored less than 50 mutations, all were included). Any variants that belonged to genes/transcripts that had since been deprecated were then excluded. Further filtering was performed based on variant call quality information available in the VCF files. Only variants with a PASS in the FILTER column and a median genotype quality greater than 20 were retained. Next, for variants with allele counts less than three, only those variants with median depth > 30 and < 500 were retained. In the case of variants with allele counts greater than three, this criterion was relaxed (median depth > 10 and < 500). However, if a variant failed these criteria but was observed in the original MutPred2 training set, the variant was retained.

Under the assumption that the development date for the original MutPred2 model was recent enough to minimize overlap with other methods' training sets, only those variants that were not present in the original MutPred2 training set were retained. Owing to the large data set size, except for MutPred2, all methods' scores were directly obtained from the dbNSFP database[43] (v3.5). In cases where the chromosomal positions in this database mapped to multiple protein positions, for simplicity, the mean of all scores was taken.

1.2.6 MutPred2.1

The above quasi-prospectively collected data set was combined with the original MutPred2 training set to create a larger training set for an updated model referred to as MutPred2.1. Apart from the training set, all aspects of this new model were identical to the original MutPred2 model, with two exceptions: (1) the number of neural networks in the ensemble was increased to 100 to ensure that all data points were sufficiently covered in each out-of-bag sample for evaluation purposes, and (2) non-pathogenic variants obtained through interspecies alignments were removed to exclude easy-to-predict variants.

1.3 Positive-unlabeled predictors for ranking mechanisms

1.3.1 Data sets

For those properties that relied on predictors developed as a part of MutPred2, an unlabeled data set was first created by randomly sampling 20 residues from each protein in MutPred2's training set and extracting the respective feature sets for each property (the same as those described in Section 1.1). Then, for each property, a final unlabeled set was created by further sampling 10,000 data points from the master set. This number ensured that a very low class prior probability (1×10^{-4}) could be estimated; i.e., if there was only one positive prediction for a given property in the unlabeled set. In the case of PTMs, only the specific modifiable residues were sampled for each PTM type. Next, 10,000 positive data points were randomly sampled for each property from their respective training data sets to ensure a balanced training set. In cases where the original training sets contained fewer than 10,000 examples, all positives were included.

For predictors where the training data and/or code were not amenable to retraining in the positive-unlabeled setting, alternative approaches were utilized to estimate class priors and specific data sets were collected for this purpose. In the case of intrinsically disordered, coiled-coil and calmodulin-binding regions, an unlabeled set of prediction scores was created by running the original predictors on all proteins in the MutPred2 training set (wild-type only) and randomly sampling 10 scores from each protein. The positive sets for intrinsic disorder and calmodulin-binding were created by including the original predictors' scores for positive data points from their original training sets. Since the training and evaluation code for coiled-coil region prediction was implemented for this study, these scores could be obtained through cross-validation. In the case of stability prediction, the unlabeled set comprised at most 10 missense mutations each from 1,500 randomly chosen proteins in the MutPred2 training set. The sequence-based prediction of stability is prone to overfitting, and therefore, a new positive set was constructed by merging the data sets from Potapov et al.[44] and Khan et al.[45] (downloaded from VariBench[46]) and filtering them against the original predictor's training set. Additionally, only those mutations with $|\Delta\Delta G| > 1$ kcal/mol were retained. Thus, the positive set comprised only mutations known to significantly impact stability, irrespective of their directionality.

1.3.2 Training and evaluation

It is important to note that, although positive data points were trained against unlabeled data points in Section 1.1, the unlabeled sets came from the same proteins as the positives and were carefully constructed to account for biases in the positive sets; e.g., removal of highly similar data points. Therefore, these predictors needed to be recast in a strictly positive-unlabeled framework, by retraining them using their positive sets against unbiased random sets of unlabeled data points (as described above).

To this end, positive-unlabeled predictors for each property were trained and evaluated using the same protocol. The AlphaMax algorithms proposed by Jain et al. assume that the underlying predictor outputs scores comparable to the un-

derlying posterior distribution.[47, 48] Therefore, for each property, an ensemble of 30 feed-forward neural networks was trained with bagging. Each network consisted of a single hidden layer with four neurons with the tansig function chosen as the activation function (for the output layer as well). As before, each network was trained on a balanced sample of positives and unlabeled data points. Feature selection was performed using a t-test at a P-value threshold of 1×10^{-4}, followed by z-score normalization and PCA to remove co-linear and nearly co-linear features (retained variance: 99%). The resilient propagation algorithm was used for training and it was stopped either after 1000 iterations or if the maximum number of validation checks was 500. In the case of stability, apart from the classification and regression predictions from the original predictor, eight additional features were included to account for possible biases and overfitting - prediction scores for the three secondary structure categories and VSL2B intrinsic disorder at the mutated position (for both the wild-type and mutated residue). All predictors were evaluated through a cross-validation procedure with the AUC measure, as described in Section 1.1.3.

1.3.3 Estimation of class prior probabilities

Due to the inherent noise in biological data sets, the noisy AlphaMax[47] algorithm was chosen to estimate class priors for the properties in MutPred2, with some exceptions (see below). This algorithm uses a two-step procedure, where AlphaMax[48] is first run to estimate an intermediate class prior value and then run on a flipped data set where the unlabeled data points are treated as positives and the labeled data points are treated as negatives. This allows for the intermediate estimation of the proportion of noise in the positive set. Finally, these values are adjusted to obtain the final estimates of class priors and noise proportions. It is important to note that although the algorithm automatically detects the inflection point in the log-likelihood curve and returns the corresponding class prior and noise estimates, this can be unreliable. Therefore, all pairs of log-likelihood curves for all properties were manually inspected and the estimated values were corrected wherever applicable. Furthermore, the estimated class prior is limited by the size of the unlabeled set; i.e., for properties with priors $< 1 \times 10^{-4}$, the log-likelihood curves were characterized by unusual spikes and/or the lack of an initial flat region. In such cases, parameters determining the points at which log-likelihood values were calculated, were changed; e.g., initial point and interval size. If these did not yield improvements in the nature of the curve, both estimates were set to 1×10^{-4}.

In the case of intrinsic disorder, calmodulin binding and coiled-coil regions, we used the same approach due to practical constraints; however, it is important to note that these models were not trained in a strictly positive-unlabeled setting. In the case of secondary structure and B-factor, it was assumed that current fractions in PDB provide reasonably accurate estimates of true prior probabilities and sophisticated approaches (such as those above) were not needed. Therefore, these fractions were recorded in 1,000 randomly selected monomers from PDB. This was repeated 10 times and the final class prior was taken to be the mean value obtained from these 10 iterations. In the case of sequence motifs, no prior

probabilities were estimated because this property did not utilize supervised learning methods.

1.3.4 Transformation to posterior distribution

The estimated class priors were combined with raw prediction scores from the positive-unlabeled predictors using the transformation described by Jain et al.[47] to obtain posterior probabilities, as follows:

$$p(Y=1|x)=\frac{\alpha(1-\alpha)}{\beta-\alpha}\left(\frac{p(S=0)}{p(S=1)}\frac{\tau(x)}{1-\tau(x)}-\frac{1-\beta}{1-\alpha}\right) \tag{3}$$

where α is the estimated class prior (the fraction of positives in the unlabeled set), β is the fraction of positives in the set labeled as positive, $p(S=0)$ and $p(S=1)$ are the fractions of unlabeled and labeled (positive) data points in the training set, respectively, and τ(x) is the raw prediction score. Here, in almost all cases, the ratio $p(S=0)/p(S=1)$ is set to one because each member of the neural network ensemble was trained on a balanced set of positive and unlabeled data. This transformation assumes that the raw prediction scores approximate the underlying posterior distribution between positive and unlabeled data as closely as possible. This assumption may not hold in practice and, as a result, can lead to posteriors outside the allowed interval between zero and one. In such cases, all values below zero were changed to zero and all values above one were changed to one.

As before, even when the problems were not strictly positive-unlabeled, this same approach was adopted for the sake of convenience; i.e., if the set of negative examples is biased, it might be beneficial to ignore them and exploit an unbiased unlabeled set. In cases where the class priors were estimated directly from real data sets (secondary structure and B-factors), posterior probabilities were obtained using a transformation for positive-negative learning[49, 50] as more appropriate:

$$p(Y=1|x)=\frac{\dfrac{\alpha\tau(x)}{p(S=1)}}{\dfrac{\alpha\tau(x)}{p(S=1)}+\dfrac{(1-\alpha)(1-\tau(x))}{p(S=0)}} \tag{4}$$

where the notation is the same as above.

2 Supplementary Notes

2.1.1 Comparison with MutPred

When evaluating the MutPred2 model on MutPred's training set in cross-validation experiments, we found that MutPred2 outperformed the original MutPred approach (Supplementary Fig. 2). This serves to confirm the utility of including molecular data through an updated feature set and algorithms for feature representation and learning. To further understand which aspects of MutPred2 have improved over MutPred, we tested two hypotheses through cross-validation on the MutPred2 training set. First, we surmised that the difference in performances of MutPred2 and MutPred could be due to differing encoding schemes and features that capture conservation in these tools (specifically, the consideration of conservation over the neighborhood of the substitution and the inclusion of unnormalized conservation scores; Supplementary Table 8). To test this hypothesis, we trained a model that only contained features derived from PSI-BLAST and conservation scores that best represented those included in the original MutPred model. This baseline model resulted in an AUC of 80.5%. We found that when neighborhood conservation was included, the AUC increased to 82.5% and that it further increased to 84.5% when unnormalized conservation scores were added. Second, we investigated whether MutPred2 benefited from an expanded set of structural and functional properties. Since these features tend to be highly correlated with conservation, we trained two models that solely utilized features derived from these properties, a model with all properties included in MutPred2 and a model with the 14 original properties from MutPred. We found that the expanded property set resulted in an improvement in performance (76.3% vs. 73.3%). Although this represents a modest improvement, we note that it is achieved despite the fact that, unlike MutPred, each built-in property predictor in MutPred2 is minimally customized and optimized for its prediction task.

2.1.2 Prospective evaluation and updated training sets

We conducted an additional evaluation of MutPred2 and other state-of-the-art methods on an independent data set collected in a manner similar to that in community-wide critical assessment experiments.[51, 52] Briefly, we allowed for pathogenic variants accumulating in HGMD over time and extracted these variants from the newer version of the database for the positive set in our evaluations. For the negative set, we sampled missense variants from gnomAD,[53] thereby creating, to the best of our knowledge, the largest independent set for the evaluation of this prediction task (19,953 pathogenic and 603,885 benign variants).

We found that on this data set, predictive performance of all methods generally decreased when compared to that in Fig. 2 (Supplementary Fig. 3a). A notable exception was FATHMM, which emerged as the top performer in terms of AUC (81.6%). MutPred2 and the original MutPred model followed with AUC values of 79.1% and 78.3%, respectively. However, at lower FPR values both MutPred2 and MutPred outperform FATHMM (Supplementary Table 11). Furthermore, we

suspected that on this large data set, performance may be driven by proteins that harbored variants from only one class (either entirely pathogenic or entirely benign). To test this, we re-evaluated these methods only on the set of those proteins that contained both classes of variants. On this data set of bi-class proteins, while MutPred2's performance was comparable with that of PolyPhen-2, it was the best performer in the low-FPR region of the ROC curve (Supplementary Fig. 3b). Interestingly, the performance of FATHMM dropped substantially, suggesting an overdependence on protein-specific features.

We note that the evaluation here differs from that in the main text in terms of: (1) the ratio of the sizes of the positive and negative sets, (2) the amount of potential noise in the negative set (gnomAD may contain undiscovered pathogenic variants), and (3) the level of stringency in creating the data set (e.g., sequence identity-based filtering of variants). These factors may explain the discrepancies between Fig. 2 and Supplementary Fig. 3.

Finally, we trained a new version of MutPred2, which we refer to as MutPred2.1, by combining this data set with the original MutPred2 training set. In order to ensure a fair comparison with other methods, we increased the number of neural networks in the ensemble to 100, and used the out-of-bag predictions extracted during training to evaluate MutPred2.1. With an AUC value of 82.8%, MutPred2.1 outperformed other methods, even in low-FPR regions of the ROC curve and on bi-class proteins, without compromising data set coverage. While our results suggest that more training data improves predictive performance of such methods, the gains are likely to be nominal, especially in light of the effort involved in retraining such models.

2.1.3 Clustering of disease categories based on affected properties

We further broke down the mutations derived from HGMD based on the diseases that they were involved in. As in previous work, we mapped general UMLS concepts to disease names and created disease subsets corresponding to 17 broad disease categories.[54] We then repeated the same enrichment analysis as that on the full data set (Online Methods; Fig. 3). As expected, metabolic disorders and the full data clustered together with respect to the pattern of enriched properties (Supplementary Fig. 6). Two broad clusters were observed when considering the diseases: a cluster with psychiatric, reproductive and respiratory diseases and a cluster with all other diseases. It is unclear why these three groups of diseases were clustered together, but it appears to be largely due to a depletion of mutations impacting metal-binding. In terms of properties, hydroxylation was singled out as an outlier, largely due to its enrichment in musculoskeletal and skin disorders. This can be explained by the propensity for collagen (frequently occurring in these organs/systems) to undergo hydroxylation at multiple positions.[55] Furthermore, there were two large sub-clusters similar to the trend observed in Fig. 3 of the main manuscript; one dominated by ordered and structural properties and the other dominated by properties related to flexibility and disorder. This second category is largely present as depletions, as in the case of the full data set.

3 Supplementary Tables

Supplementary Table 1 | Residue-level properties included in MutPred2.

Structural property	Functional property
Intrinsic disorder	Catalytic
B-factor	Calmodulin binding
Relative solvent accessibility	DNA binding
Helix	RNA binding
Strand	Protein-protein interaction (PPI)
Loop	PPI hotspot
Signal peptide and topology (N-terminus, helix, C-terminus, cleavage site)	Molecular Recognition Feature (MoRF)
Transmembrane and topology (Non-cytoplasmic loop, transmembrane helix, cytoplasmic loop)	Allosteric
Coiled-coil region	Metal binding (11: cadmium, calcium, cobalt, copper, iron, magnesium, manganese, nickel, potassium, sodium, zinc)
Disulfide linkage	Post-translational modification (22: acetylation, ADP-ribosylation, amidation, C-linked glycosylation, carboxylation, farnesylation, geranylgeranylation, GPI-anchor amidation, hydroxylation, methylation, myristoylation, N-linked glycosylation, N-terminal acetylation, O-linked glycosylation, palmitoylation, phosphorylation, proteolytic cleavage, pyrrolidone carboxylic acid, sulfation, SUMOylation, ubiquitylation)
Stability	Sequence motif

Supplementary Table 2 | Summary of training data sets that were derived from protein structure data.

Property	Structures	Chains
Relative Solvent Accesibility	215	215
Catalytic	707	721
DNA	132	138
RNA	130	195
PPI	86	112
PPI hotspot	56	78
MoRF	453	456
Cadmium	188	199
Calcium	1,071	1,092
Cobalt	154	156
Copper	103	105
Iron	185	187
Magnesium	1,313	1,348
Manganese	362	365
Nickel	249	254
Potassium	282	287
Sodium	945	961
Zinc	1,287	1,307

Supplementary Table 3 | Summary of different structural property predictors.

Structural properties	Data source (Reference)	Classes	Data set size after re-dundancy removal		Final model
			Positives	Negatives	
Relative solvent accessibility	1	Exposed/buried	28,050	22,799	Neural net-work ensem-ble
Signal / trans-membrane (TM) topology	2	Signal peptide (N-terminus)	9,069	765,589	8-output neu-ral network ensemble
		Signal helix	14,294		
		Signal peptide (C-terminus)	8,408		
		Signal cleav-age	1,320		
		TM (inside)	40,221		
		TM helix	29,241		
		TM (outside)	48,821		
Coiled-coil re-gion	3	Coiled coil / not coiled coil	64,431	179,593	Hidden Mar-kov model

Supplementary Table 4 | Summary of different functional property predictors

Functional properties	Data source (Reference)	Classes	Data set size after redundancy removal		Final model
			Positives	Negatives	
Catalytic	4	Catalytic/not catalytic	2,162	13,194	Logistic regression ensemble
DNA-binding	4	Binding/non-binding	3,668	2,890	Logistic regression
RNA-binding	5	Binding/non-binding	7,776	4,900	Neural network ensemble
Protein-protein interaction (PPI)	4	Binding/non-binding	4,283	8,706	Neural network ensemble
PPI hotspot	6	Hotspot/non-hotspot	109	350	Logistic regression ensemble
MoRF	7	MoRF/non-MoRF	5,689	271,202	Random forest
Allosteric	8	Allosteric/not allosteric	1,446	107,593	Random forest
Metal-binding	4	Cadmium	981	984	Neural network ensemble
		Calcium	4,406	4,724	Random forest
		Cobalt	510	526	Random forest
		Copper	361	427	Neural network ensemble
		Iron	726	774	Neural network ensemble
		Magnesium	2,960	3,235	Random forest
		Manganese	1,267	1,322	Neural network ensemble
		Nickel	647	657	Random forest
		Potassium	940	975	Random forest
		Sodium	2,695	2,737	Random forest
		Zinc	5,237	5,650	Random forest

Supplementary Table 5 | Performance values for property predictors.

Predictor	AUC	*sn* at *sp* ≈ 90%	*sn* at *sp* ≈ 95%	*sn* at *sp* ≈ 99%
Relative solvent accessibil-ity	87.51	64.68	49.19	20.09
Signal peptide (N-terminus)	97.35	95.21	93.40	89.49
Signal helix	98.89	98.25	97.49	93.20
Signal peptide (C-terminus)	97.69	96.69	94.75	83.33
Signal cleavage	83.84	80.83	68.71	53.41
Cytoplasmic loop	78.27	49.68	40.53	22.33
Transmembrane region	98.16	96.70	95.04	83.40
Non-cytoplasmic loop	71.83	34.47	24.31	12.91
Non-transmembrane	83.18	32.18	20.72	1.23
Coiled coil	95.37	90.85	81.47	76.51
Catalytic residue	90.07	67.81	48.24	14.29
DNA-binding	80.82	44.55	28.52	6.24
RNA-binding	79.33	43.69	29.63	11.86
PPI residue	70.10	26.99	14.52	3.95
PPI hotspot	66.65	27.52	7.34	2.75
MoRF	73.15	35.77	24.03	8.07
Allosteric site	73.41	29.94	18.12	3.73
Cadmium-binding	62.25	21.41	15.60	4.38
Calcium-binding	73.30	39.70	26.74	8.49
Cobalt-binding	77.34	47.84	34.90	10.98
Copper-binding	81.87	50.42	36.84	20.22
Iron-binding	89.15	73.55	49.45	11.98
Magnesium-binding	77.18	45.41	32.94	14.86
Manganese-binding	83.71	54.22	34.81	16.73
Nickel-binding	70.77	39.88	27.05	7.57
Potassium-binding	65.51	21.28	12.66	2.77
Sodium-binding	64.03	22.12	12.88	4.04
Zinc-binding	83.01	60.24	47.87	24.21

Supplementary Table 6 | Summary of the results for the positive-unlabeled property predictors used to infer molecular mechanisms of disease. Sizes of the training sets are shown along with performance values estimated through cross-validation. The last two columns show the parameters estimated for each property using these predictors (α is the estimated class prior and β is the estimated fraction of positives in the positive set). For PTMs, prior probabilities were calculated only on the modifiable residues for each PTM. Prior probabilities for helix, strand, loop and B-factor were calculated directly from PDB and were found to be 0.2767, 0.1804, 0.3102 and 0.1546, respectively.

Property	Number of sites		AUC	α	β
	Positive	Unlabeled			
Intrinsic disorder	51,954	298,210	NA	0.2327	0.9309
Relative solvent accessibility	10,000	10,000	87.12	0.0491	0.9809
N-terminal signal	9,069	10,000	99.26	0.0001	0.9999
Signal helix	10,000	10,000	99.26	0.0001	0.9999
C-terminal signal	8,408	10,000	99.07	0.0001	0.9999
Signal cleavage	1,320	10,000	98.87	0.0001	0.9999
Non-cytoplasmic loop	10,000	10,000	86.40	0.1081	0.9821
Transmembrane region	10,000	10,000	97.51	0.0001	0.9999
Cytoplasmic loop	10,000	10,000	76.40	0.0294	0.9806
Coiled coil	64,431	298,210	NA	0.0291	0.9709
Catalytic site	2,162	10,000	97.22	0.0103	0.9999
Calmodulin binding	924	298,210	NA	0.0778	0.9723
DNA binding	1,446	10,000	90.55	0.0198	0.9705
RNA binding	3,668	10,000	92.04	0.0001	0.9999
PPI residue	7,776	10,000	84.02	0.1180	0.9823
PPI hotspot	4,283	10,000	80.51	0.0688	0.9813
MoRF	109	10,000	80.04	0.1232	0.9473
Allosteric site	5,689	10,000	85.66	0.0393	0.9807
Cadmium binding	981	10,000	94.25	0.0001	0.9999
Calcium binding	4,406	10,000	93.76	0.0001	0.9999
Cobalt binding	510	10,000	96.14	0.0001	0.9799
Copper binding	361	10,000	97.46	0.0099	0.9801
Iron binding	726	10,000	98.43	0.0001	0.9999
Magnesium binding	2,960	10,000	93.19	0.0001	0.9999
Manganese binding	1,267	10,000	96.77	0.0197	0.9803
Nickel binding	647	10,000	96.04	0.0193	0.9607
Potassium binding	940	10,000	89.31	0.0197	0.9803
Sodium binding	2,695	10,000	87.09	0.0295	0.9805
Zinc binding	5,237	10,000	96.70	0.0201	0.9999
Acetylation	6,848	10,000	72.08	0.1371	0.9137

ADP-ribosylation	108	10,000	76.29	0.1090	0.9911
Amidation (motif)	210	609	94.02	0.0197	0.9803
Amidation (non-motif)	247	10,000	98.30	0.0001	0.9999
C-linked glycosylation	32	7,651	99.43	0.0001	0.9999
Carboxylation	112	10,000	96.50	0.0001	0.9399
Disulfide linkage	9,736	10,000	85.40	0.0974	0.9728
Farnesylation	41	10,000	97.38	0.0001	0.9399
Geranylgeranylation	30	10,000	98.59	0.0001	0.9999
GPI anchor amidation	84	10,000	98.10	0.0197	0.9803
Hydroxylation	214	10,000	94.77	0.0001	0.9599
Methylation (K)	303	10,000	72.77	0.0650	0.8130
Methylation (R)	325	10,000	80.49	0.0142	0.7041
Myristoylation	99	10,000	99.40	0.0001	0.9799
N-linked glycosylation (motif)	10,000	2,757	80.18	0.0681	0.9720
N-linked glycosylation (non-motif)	719	10,000	86.77	0.0001	0.8599
N-terminal acetylation (A)	527	10,000	100.00	0.0001	0.9999
N-terminal acetylation (G)	21	10,000	99.22	0.0001	0.9999
N-terminal acetylation (M)	334	10,000	98.04	0.0197	0.9803
N-terminal acetylation (S)	345	10,000	99.96	0.0001	0.9999
N-terminal acetylation (T)	83	10,000	99.90	0.0001	0.9999
O-linked glycosylation (S)	755	10,000	85.03	0.0283	0.9416
O-linked glycosylation (T)	672	10,000	87.36	0.0544	0.9053
Palmitoylation	245	10,000	91.30	0.0001	0.9699
Phosphorylation (S)	10,000	10,000	82.09	0.0876	0.9725
Phosphorylation (T)	10,000	10,000	80.42	0.0584	0.9717
Phosphorylation (Y)	10,000	10,000	75.77	0.1462	0.9744
Proteolytic cleavage	997	10,000	79.91	0.0172	0.8624
Pyrrolidone carboxylic acid	275	10,000	90.38	0.0389	0.9711
Sulfation	121	10,000	95.68	0.0393	0.9807
SUMOylation (motif)	523	1,342	79.61	0.1031	0.9372
SUMOylation (non-motif)	221	10,000	68.06	0.1291	0.8606
Ubiquitylation	1,092	10,000	63.54	0.0947	0.7284
Stability	817	10,045	84.87	0.0291	0.9709

Supplementary Table 7 | Performance values for different combinations of training and test sets. AUC values from 10-fold cross-validation are shown. Colored cells correspond to the best performing model (training set) for a given test set.

**TRAINING**				**TEST**									
**Disease**		**Non-disease**											
**Data set**	**Size**	**Data set**	**Size**	**AD vs. AN**	**AD vs. CN**	**AD vs. RN**	**AD vs. dbS**	**AD vs. SP**	**AD vs. Ho**	**HG vs. SP**	**DWF vs. NWF**	**DWF vs. CN**	**DWF vs. RN**
All **(AD)**	53,180	All **(AN)**	206,946	87.7	92.9	87.4	87.8	88.2	95.6	88.6	80.2	87.1	78.8
		Common **(CN)**	17,634	86.5	92.8	86.5	86.5	87.7	95.9	88.1	79.6	87.2	78.2
		Rare only **(RN)**	90,214	86.7	92.7	88.2	86.9	87.0	95.0	87.3	79.5	85.9	78.3
		dbSNP **(dbS)**	190,509	87.7	92.8	87.5	87.8	88.1	94.8	88.5	80.1	86.9	78.8
		SwissProt **(SP)**	33,042	86.6	92.4	85.8	86.5	88.3	95.4	88.8	79.4	87.0	77.9
		Homology **(Ho)**	6,193	75.2	82.8	73.0	74.5	78.9	99.4	79.2	71.5	79.9	69.8
HGMD **(HG)**	50,949	SP	33,042	86.5	92.2	85.6	86.4	88.3	95.3	88.7	79.3	86.9	77.9
With allele frequency **(DWF)**	7,856	With allele frequency **(NWF)**	107,848	85.1	91.4	84.0	85.0	86.5	95.3	86.9	80.1	87.6	78.7
		CN	17,634	83.5	90.9	81.5	83.2	85.9	95.1	86.3	79.4	88.0	77.8
		RN	90,214	85.1	91.1	84.1	85.1	86.3	94.8	86.6	80.2	87.0	78.8

Supplementary Table 8 | Top 50 features (~5% of all significant features) in MutPred2 as determined by Wilcoxon rank sum tests. Since P-values were extremely small (and identical), the features are ranked by the absolute value of the z-statistic. As shown, these features correspond to one of the nine conservation scores in AL2CO or the column frequency of the reference or alternate residue in alignments (at or in the neighborhood of the substitution position).

Feature	Window length	|*z*-statistic|
Mean normalized unweighted entropy (Vertebrates)	1	180.60
Mean normalized independent count entropy (Vertebrates)	1	179.38
Mean unnormalized unweighted entropy (Vertebrates)	1	178.79
Mean normalized unweighted sum-of-pairs (Vertebrates)	1	178.33
Mean normalized independent count variance (Vertebrates)	1	176.05
Mean normalized independent count sum-of-pairs (Vertebrates)	1	175.64
Mean unnormalized unweighted sum-of-pairs (Vertebrates)	1	175.56
Mean unnormalized independent count entropy (Vertebrates)	1	175.35
Mean normalized Henikoff entropy (Vertebrates)	1	175.12
Mean unnormalized Henikoff entropy (Vertebrates)	1	173.84
Mean unnormalized independent count sum-of-pairs (Vertebrates)	1	173.14
Mean unnormalized independent count variance (Vertebrates)	1	172.55
Mean normalized Henikoff sum-of-pairs (Vertebrates)	1	172.15
Mean unnormalized unweighted entropy (Mammals)	1	171.97
Gapped column frequency of alternate residue (Vertebrates)	1	171.88
Mean unnormalized Henikoff sum-of-pairs (Vertebrates)	1	169.66
Mean unnormalized independent count entropy (Mammals)	1	168.29
Mean unnormalized Henikoff entropy (Mammals)	1	167.26
Mean normalized unweighted variance (Vertebrates)	1	166.42
Mean unnormalized unweighted variance (Vertebrates)	1	166.29
Mean unnormalized unweighted sum-of-pairs (Mammals)	1	163.18
Mean unnormalized Henikoff variance (Vertebrates)	1	162.32
Mean normalized Henikoff variance (Vertebrates)	1	162.27
Mean normalized independent count entropy (Mammals)	1	162.02
Mean PSI-BLAST information per position (Mean)	1	161.93
Mean normalized unweighted entropy (Mammals)	1	160.91
Mean normalized independent count sum-of-pairs (Mammals)	1	160.89
Mean unnormalized independent count sum-of-pairs (Mammals)	1	160.00
Mean unnormalized independent count variance (Mammals)	1	159.63
Mean normalized unweighted sum-of-pairs (Mammals)	1	159.46
Mean normalized independent count variance (Mammals)	1	158.82
Gapped column frequency of alternate residue (Mammals)	1	158.39
Mean unnormalized Henikoff sum-of-pairs (Mammals)	1	157.55
Gapped column frequency of reference residue (Vertebrates)	1	157.23

Mean normalized Henikoff entropy (Mammals)	1	155.25
Mean unnormalized independent count variance (Vertebrates)	5	154.31
Mean unnormalized independent count sum-of-pairs (Vertebrates)	5	154.05
Mean unnormalized independent count sum-of-pairs (Vertebrates)	11	153.95
Mean unnormalized independent count variance (Vertebrates)	11	153.40
Mean normalized Henikoff sum-of-pairs (Mammals)	1	153.06
Mean unnormalized unweighted variance (Mammals)	1	152.30
Ungapped column frequency of alternate residue (Vertebrates)	1	151.91
Ungapped column frequency of reference residue (Vertebrates)	1	150.92
Mean unnormalized unweighted sum-of-pairs (Vertebrates)	5	150.17
Mean unnormalized independent count variance (Mammals)	5	149.45
Mean unnormalized independent count sum-of-pairs (Vertebrates)	21	148.75
Mean unnormalized unweighted variance (Vertebrates)	5	148.56
Mean unnormalized independent count sum-of-pairs (Mammals)	5	148.46
Mean unnormalized independent count variance (Mammals)	11	147.96
Mean unnormalized independent count sum-of-pairs (Mammals)	11	147.75

Supplementary Table 9 | Top 25 features corresponding to loss or gain of structural and functional properties in MutPred2, ranked by the absolute value of the z-statistic, as determined by Wilcoxon rank sum tests. The highest z-statistic value here is lower than those in Supplementary Table 8, suggesting that conservation-based features are more discriminative than these 53 properties.

Feature	Window length	|z-statistic|
Transmembrane region (gain)	11	105.43
Transmembrane region (loss)	11	104.52
Allosteric site (gain)	11	88.39
Cytoplasmic loop (loss)	11	78.02
Allosteric site (loss)	11	77.79
Cytoplasmic loop (gain)	11	77.14
N-terminal signal (loss)	11	72.29
Signal cleavage (loss)	11	62.56
N-terminal signal (gain)	11	61.89
Signal helix (gain)	11	61.29
Signal helix (loss)	11	60.82
Signal cleavage (gain)	11	54.97
Strand (loss)	11	42.80
Strand (gain)	11	42.01
C-terminal signal (loss)	11	39.71
C-terminal signal (gain)	11	39.13
Catalytic site (loss)	11	35.50
ADP-ribosylation (loss)	11	33.94
Manganese-binding (loss)	11	33.86
B-factor (loss)	11	33.50
Intrinsic disorder (gain)	11	33.49
Relative solvent accessibility (gain)	11	32.58
Carboxylation (loss)	11	31.63
Sulfation (gain)	11	31.60
PPI residue (loss)	11	31.19

Supplementary Table 10 | Detailed performance values for MutPred2 and other methods on the independent test set, filtered against these methods at 50% sequence identity. Sensitivity (Sens.), precision (Prec.) and balanced accuracy (Bal. acc.) were recorded at specificity (Spec.) value of 95%. NA denotes cases where specificity values were not available at this threshold.

**Full set (343 pathogenic and 137 benign)**							
**Predictor**	**Coverage**		**AUC**	**Spec.**	**Sens.**	**Prec.**	**Bal. acc.**
	**Pathogenic**	**Benign**					
**MutPred2**	100.0	100.0	84.9	95.6	42.3	96.0	69.0
**MutPred**	100.0	100.0	80.1	95.6	30.6	94.6	63.1
**PolyPhen2 (HumVar)**	100.0	100.0	77.6	96.4	12.2	89.4	54.3
**FATHMM**	87.5	91.2	53.7	95.0	8.0	40.0	51.5
**SNPs&GO**	100.0	100.0	80.8	95.6	35.9	95.4	65.7
**SIFT**	94.5	84.7	82.9	95.1	38.8	73.8	66.9
**MutationTaster2**	97.7	98.5	69.7	NA	NA	NA	NA
**CADD**	99.4	100.0	83.8	95.6	28.2	94.1	61.9
**GERP++**	99.4	100.0	71.4	96.4	5.3	78.3	50.8
**PhyloP**	99.4	100.0	67.1	NA	NA	NA	NA
**Fully covered subset (285 pathogenic and 107 benign)**							
**Predictor**	**Coverage**		**AUC**	**Spec.**	**Sens.**	**Prec.**	**Bal. acc.**
	**Pathogenic**	**Benign**					
**MutPred2**	100.0	100.0	87.1	95.3	43.5	96.1	69.4
**MutPred**	100.0	100.0	82.3	95.3	39.3	95.7	67.3
**PolyPhen2 (HumVar)**	100.0	100.0	79.6	95.3	12.3	87.5	53.8
**FATHMM**	100.0	100.0	53.3	95.1	9.4	41.7	52.2
**SNPs&GO**	100.0	100.0	81.7	95.3	34.7	95.2	65.0
**SIFT**	100.0	100.0	82.6	95.1	36.5	73.6	65.8
**MutationTaster2**	100.0	100.0	72.4	NA	NA	NA	NA
**CADD**	100.0	100.0	83.9	95.3	27.4	94.0	61.4
**GERP++**	100.0	100.0	76.8	95.3	9.1	83.9	52.2
**PhyloP**	100.0	100.0	69.1	NA	NA	NA	NA

Supplementary Table 11 | Detailed performance values for MutPred2 and other methods on the independent test set, filtered against these methods at 80% sequence identity. Sensitivity (Sens.), precision (Prec.) and balanced accuracy (Bal. acc.) were recorded at specificity (Spec.) value of 95%. NA denotes cases where specificity values were not available at this threshold.

**Full set (700 pathogenic and 282 benign)**							
**Predictor**	**Coverage**		**AUC**	**Spec.**	**Sens.**	**Prec.**	**Bal. acc.**
	**Pathogenic**	**Benign**					
**MutPred2**	100.0	100.0	84.8	95.0	44.4	95.7	69.7
**MutPred**	100.0	100.0	79.7	95.0	37.3	94.9	66.2
**PolyPhen2 (HumVar)**	100.0	99.7	79.4	95.0	26.6	93.0	60.8
**FATHMM**	91.0	93.3	56.6	95.1	9.1	43.6	52.1
**SNPs&GO**	100.0	100.0	80.3	95.0	39.4	95.2	67.2
**SIFT**	94.1	81.9	79.2	95.1	29.9	68.3	62.5
**MutationTaster2**	98.6	98.6	70.4	NA	NA	NA	NA
**CADD**	99.4	99.7	83.4	95.0	28.7	93.5	61.9
**GERP++**	99.4	99.3	70.0	95.0	6.6	76.7	50.8
**PhyloP**	99.4	99.7	66.3	95.0	9.2	82.1	52.1
**Fully covered subset (602 pathogenic and 215 benign)**							
**Predictor**	**Coverage**		**AUC**	**Spec.**	**Sens.**	**Prec.**	**Bal. acc.**
	**Pathogenic**	**Benign**					
**MutPred2**	100.0	100.0	86.7	95.4	46.8	96.6	71.1
**MutPred**	100.0	100.0	81.9	95.4	41.2	96.1	68.3
**PolyPhen2 (HumVar)**	100.0	100.0	81.0	97.2	17.4	94.6	57.3
**FATHMM**	100.0	100.0	56.6	95.0	11.6	45.5	53.3
**SNPs&GO**	100.0	100.0	81.8	95.4	38.0	95.8	66.7
**SIFT**	100.0	100.0	79.5	95.0	31.6	69.4	63.3
**MutationTaster2**	100.0	100.0	73.0	NA	NA	NA	NA
**CADD**	100.0	100.0	84.1	95.4	30.2	94.8	62.8
**GERP++**	100.0	100.0	75.2	95.4	12.0	87.8	53.7
**PhyloP**	100.0	100.0	69.4	95.4	9.1	84.6	52.2

Supplementary Table 12 | Detailed performance values for MutPred2, MutPred2.1 and other methods on the newer and larger prospectively collected independent test set, Sensitivity (Sens.), precision (Prec.) and F-score (F1) were recorded at specificity (Spec.) value of 95%. NA denotes cases where specificity values were not available at this threshold.

Full set (19,953 pathogenic and 603,885 benign)							
**Predictor**	**Coverage**		**AUC**	**Spec.**	**Sens.**	**Prec.**	**F1**
	**Pathogenic**	**Benign**					
**MutPred2.1**	100.0	100.0	82.8	95.4	34.2	19.8	25.1
**MutPred2.0**	100.0	100.0	79.1	95.6	28.9	17.9	22.1
**MutPred**	87.5	77.9	78.3	95.3	28.2	18.2	22.2
**PolyPhen2 (HumVar)**	99.7	97.0	77.2	97.1	15.0	14.8	14.9
**FATHMM**	97.9	88.6	81.4	95.9	22.7	99.3	37.0
**SIFT**	97.3	96.6	75.9	95.0	23.0	99.3	37.4
**MutationTaster2**	99.3	97.5	63.5	NA	NA	NA	NA
**CADD**	99.9	98.0	75.4	96.5	10.8	9.3	10.0
**GERP++**	99.9	98.0	65.3	97.2	3.9	4.5	4.2
**PhyloP100**	99.9	98.0	72.7	96.4	11.1	9.4	10.2
**Fully covered subset (16,932 pathogenic and 417,403 benign)**							
**Predictor**	**Coverage**		**AUC**	**Spec.**	**Sens.**	**Prec.**	**F1**
	**Pathogenic**	**Benign**					
**MutPred2.1**	100.0	100.0	83.3	95.5	33.8	23.4	27.6
**MutPred2.0**	100.0	100.0	80.3	95.2	30.8	20.7	24.8
**MutPred**	100.0	100.0	77.8	95.2	28.2	19.1	22.8
**PolyPhen2 (HumVar)**	100.0	100.0	77.9	96.8	15.9	16.9	16.4
**FATHMM**	100.0	100.0	82.5	95.2	27.1	99.3	42.6
**SIFT**	100.0	100.0	76.9	95.1	25.1	99.2	40.1
**MutationTaster2**	100.0	100.0	63.6	NA	NA	NA	NA
**CADD**	100.0	100.0	76.3	96.0	12.6	11.3	11.9
**GERP++**	100.0	100.0	65.5	97.1	4.0	5.3	4.5
**PhyloP100**	100.0	100.0	73.4	96.3	11.5	11.1	11.3

Supplementary Table 13 | Mutations selected for experimental validation with minor allele frequencies (MAFs) from gnomAD. NA represents the lack of allele frequency information in gnomAD and other sources. The Type column shows the source data set for the given mutation.

Protein	Mutation	Type	gnomAD MAF
STXBP1	R551C	ASD	NA
	G544D	ASD; Early infantile epileptic encephalopathy	NA
	G561R	SNP	3.98×10^{-6}
	A113T	SNP	7.07×10^{-6}
	V84I	SNP	5.76×10^{-4}
ZBTB18	R486G	ASD	NA
	G416R	SNP	2.00×10^{-4}*
	A502T	SNP	4.03×10^{-6}
	T507A	SNP	8.11×10^{-6}
DNMT3A	R635W	ASD	6.35×10^{-5}

*MAF unavailable in gnomAD but available in 1000 Genomes Project

4 Supplementary Figures

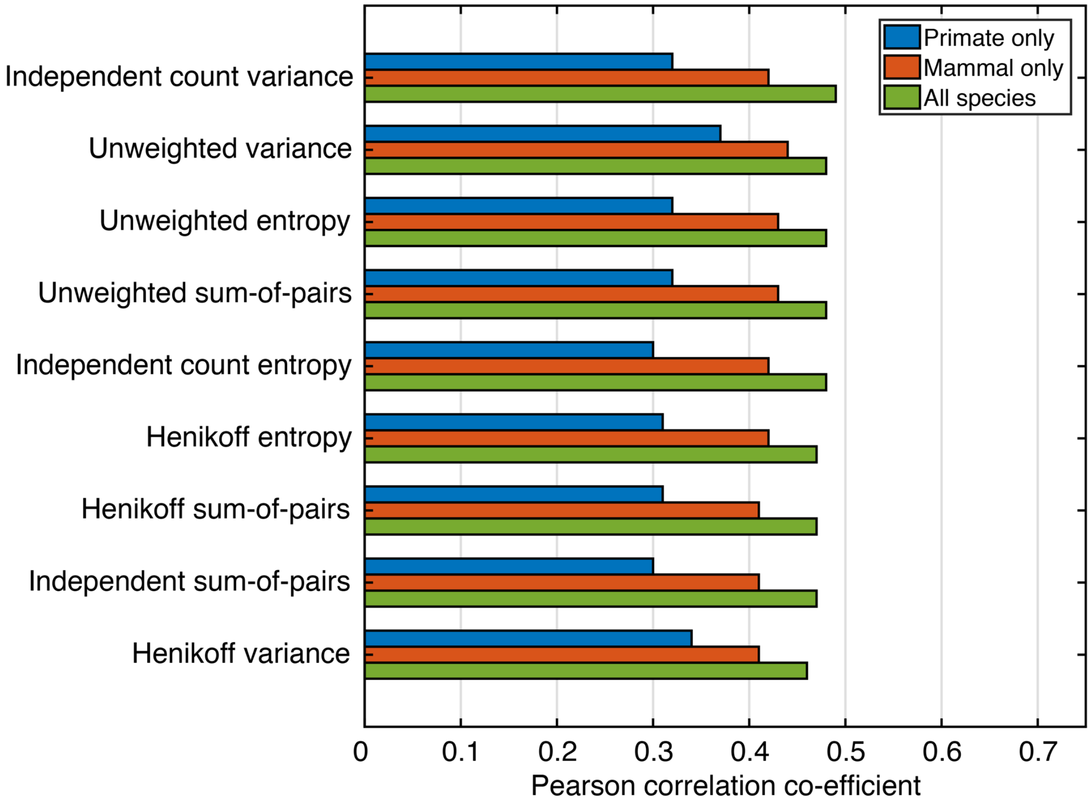

Supplementary Figure 1 | Performance values for the predictors of conservation scores. Evaluations were performed using 10-fold cross-validation on 444,660 positions sampled from 29,644 UCSC multiple sequence alignments. Since these predictions were made from PSI-BLAST PSSMs built on the NCBI nr database, correlations improved with the addition of more species to the alignment, as expected. Evaluations for alignment column residue frequency predictions resulted in high correlations in all cases (> 0.85), owing to their sparseness, and are not shown.

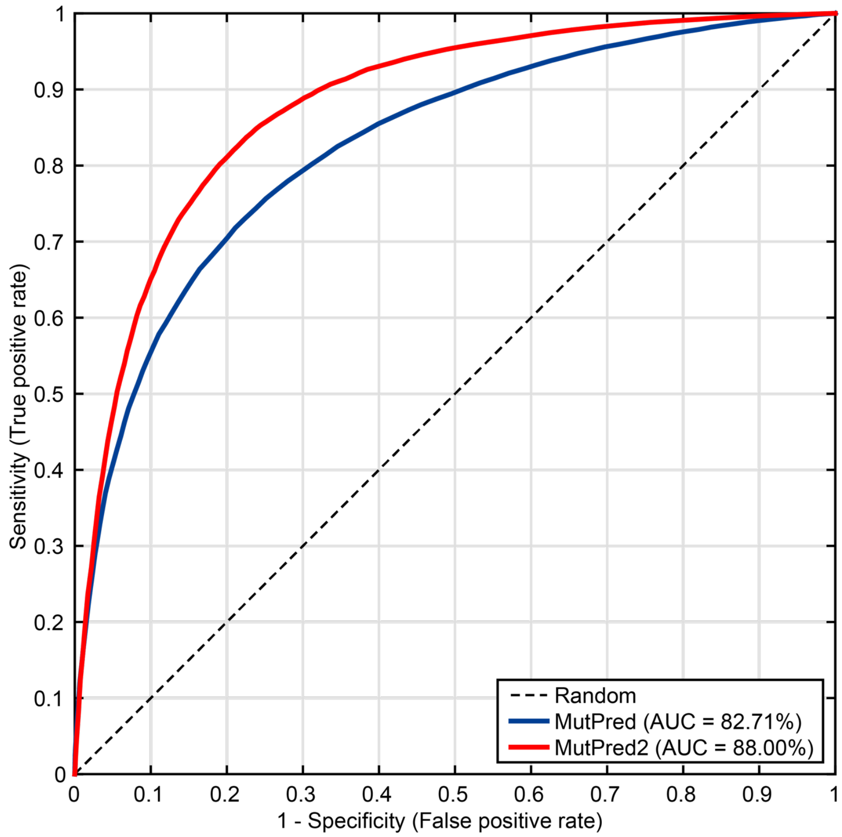

Supplementary Figure 2 | ROC curves for MutPred (v1.2) and MutPred2 in cross-validation experiments on MutPred's training set. This data set consisted of 39,115 pathogenic and 26,051 putatively benign amino acid substitutions. The cross-validation procedure ensured that variants from the same protein were either entirely in the training set or in the test set.

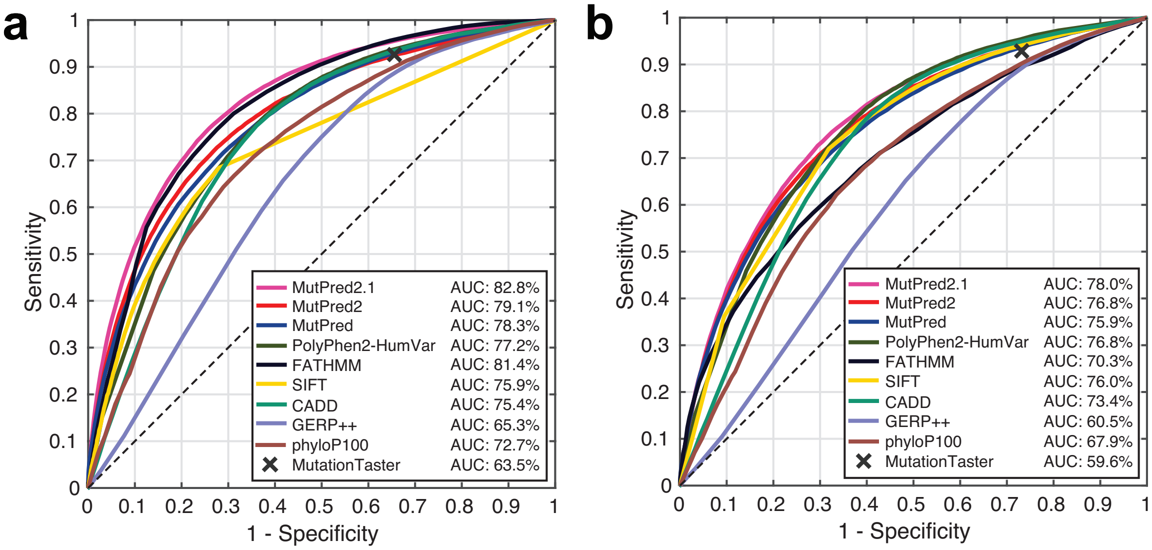

Supplementary Figure 3 | ROC curves on a larger independent test set, obtained from a newer version of HGMD and gnomAD. MutationTaster2 only returns a value of zero or one and therefore its performance is plotted as a single point (X). Since some tools could not assign scores to all variants, each tool's performance was evaluated on a slightly different subset of the data set. Detailed performance measures in this scenario as well as on the subset of the variants covered by all methods are shown in Supplementary Table 11.

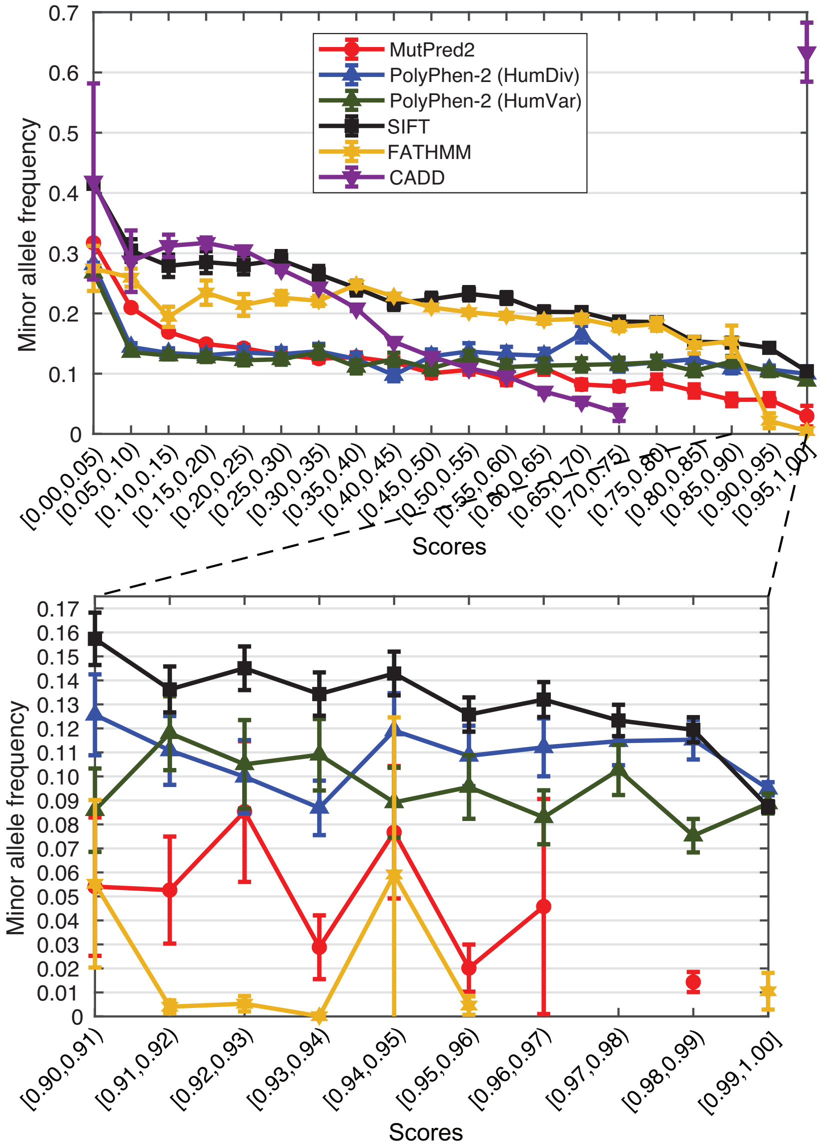

Supplementary Figure 4 | Mean minor allele frequencies (MAFs) as a function of MutPred2 scores for the 10 apparently healthy individuals' genomes. Error bars represent the standard errors of the means, estimated by dividing the standard deviation in each bin by the square root of 10. Global MAFs from the 1000 Genomes Project were used. The lower panel shows an expanded version of the high-scoring region of the distribution. Both PolyPhen-2 models show relatively flatter score distributions in low-MAF regions. FATHMM is relatively flat but shows a sharp decrease in MAF for extremely high-scoring variants. MutPred2 score distributions negatively correlate with MAFs; rarer variants are scored higher. While SIFT also negatively correlates with allele frequency, MAFs for SIFT are higher on average even in high-scoring regions (common variants also

scored highly).

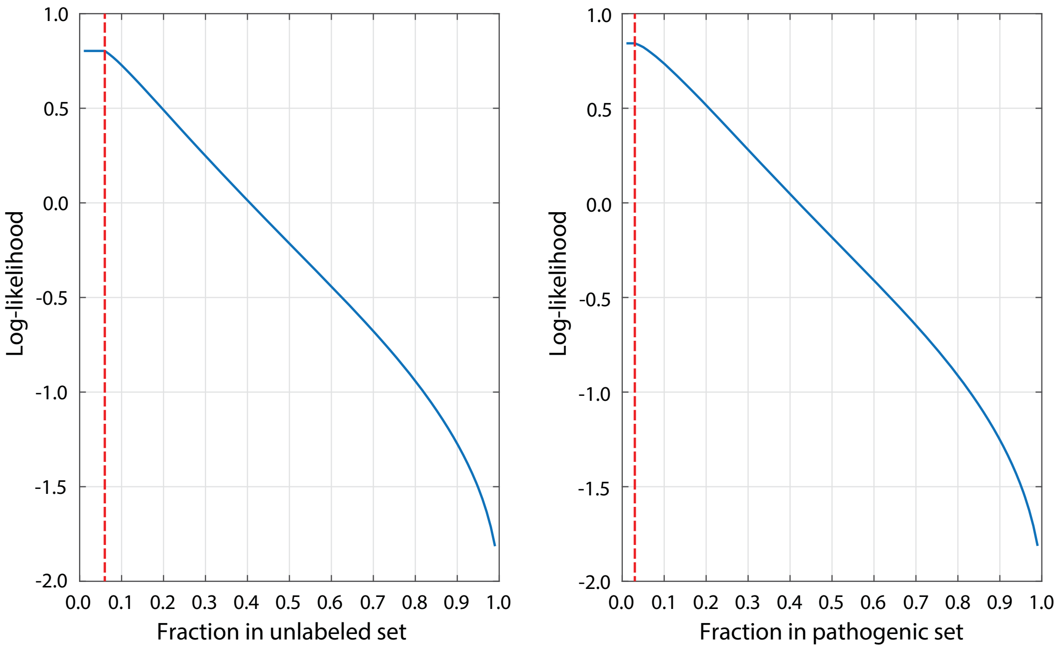

Supplementary Figure 5 | Estimation of proportions. Log-likelihood curves (blue) produced by the AlphaMax algorithm.[47, 48] The vertical red lines give estimates of the proportion of variants labeled as pathogenic in unlabeled data (left) and the proportion of unlabeled variants in the data set labeled as pathogenic (right). The proportions of pathogenic variants in the unlabeled set and the proportion of neutral variants in the pathogenic set were then derived as proposed by Jain et al. [47, 48] These proportions were used to transform MutPred2 scores on the 10 genomes to posterior probabilities. The fraction of pathogenic variants in each genome was obtained by taking the empirical mean of these posterior probabilities.

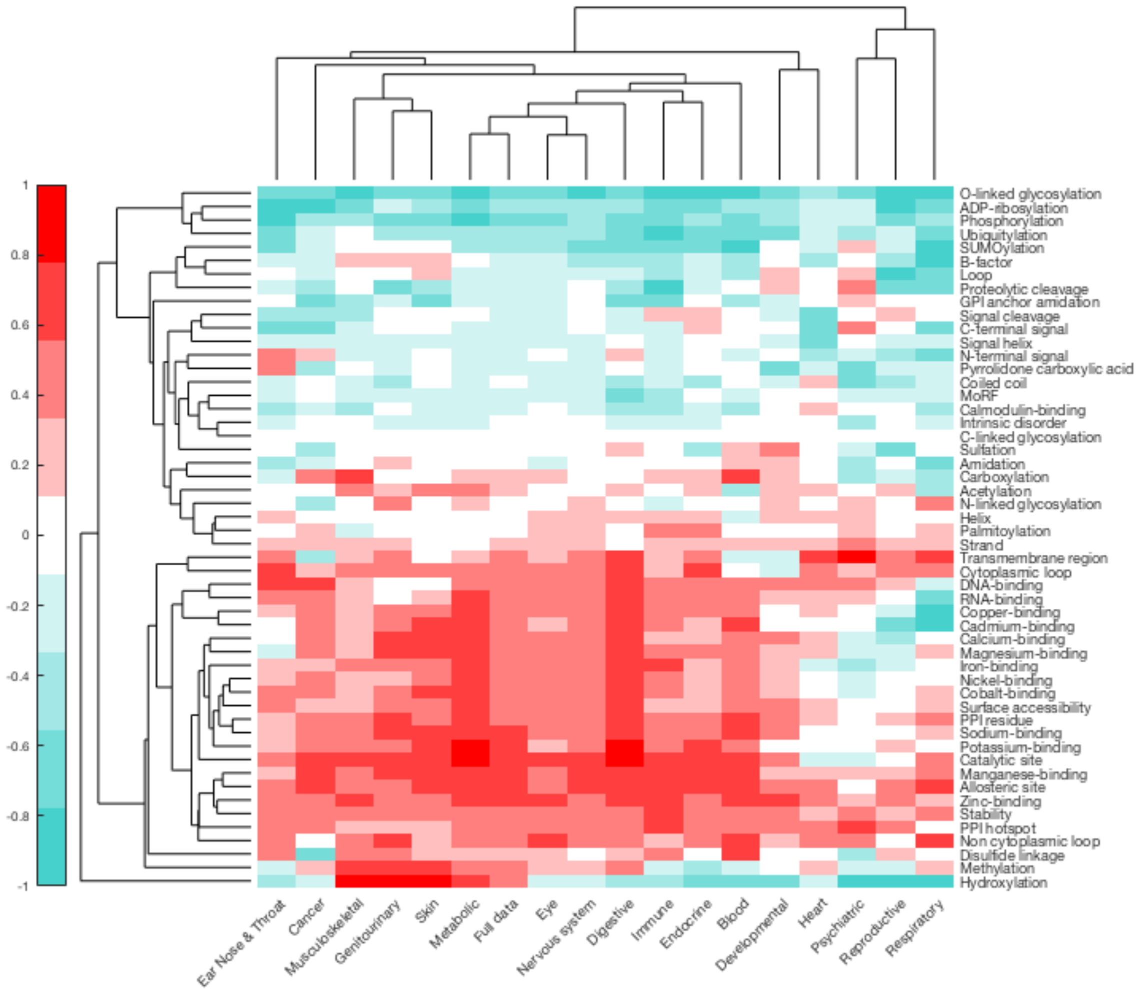

Supplementary Figure 6 | Clustergram of molecular mechanisms in different disease groups found in the Human Gene Mutation Database (HGMD). Unified Medical Language System (UMLS) concepts were used to group diseases (and their substitutions) together into one of 17 categories. Red color indicates enrichment and turquoise color indicates depletion.

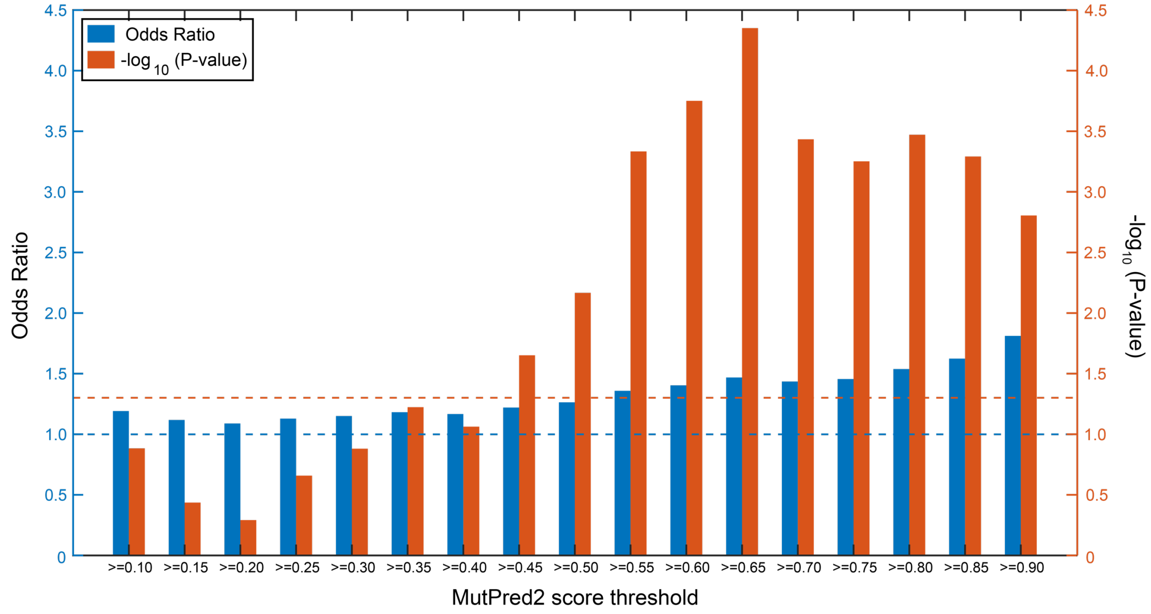

Supplementary Figure 7 | Odds ratio of the pathogenic variants predicted in neurodevelopmental case and control sets using MutPred2, as a function of pathogenicity score thresholds. For a given score threshold, the number of predicted pathogenic and benign variants in cases and controls were calculated. An odds ratio value greater than one suggests that predicted pathogenic variants are enriched in cases, at the given threshold. P-values were calculated using a two-tailed Fisher's exact test and adjusted using the Benjamini-Hochberg correction for multiple hypothesis testing. The blue dashed line corresponds to the baseline odds ratio of one and the red dashed line corresponds to the P-value threshold of 0.05. Only those genes exclusively appearing in either the case or control set were considered for this analysis, resulting in 2,986 and 844 mutations from the case and control sets, respectively. Similar trends were observed when the full data set was considered.

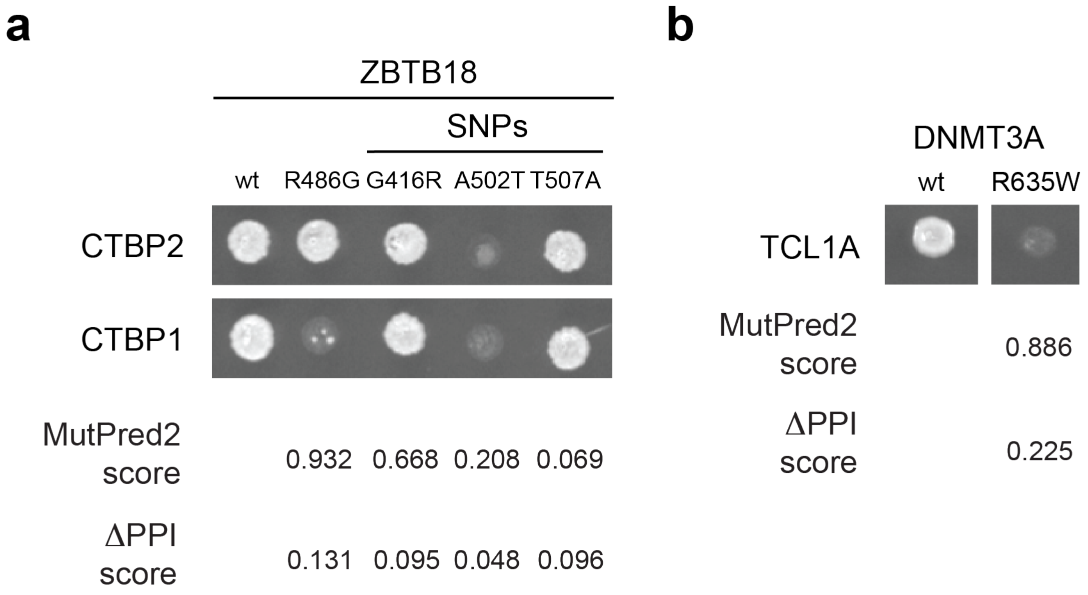

Supplementary Figure 8 | Experimental validation of the impact of mutations on protein-protein interactions using the yeast two-hybrid (Y2H) system. Representative images of the Sc-Leu-3AT selection plates with the interaction profiles of **(a)** ZBTB18 against CTBP1 and CTBP2, and **(b)** DNMT3A against TCL1A. The de novo mutation R486G in ZBTB18 was detected by exome sequencing in the individual with intellectual disability,[56] and is shown to disrupt the interaction with CTBP1 only. One of the SNPs, A502T also disrupts interaction, but with both partners. It is possible that this SNP may be an as yet undiscovered pathogenic variant for neurodevelopmental or other human disease. The de novo mutation R635W in DNMT3A was detected in the individual with autism using whole genome sequencing.[57] It disrupts the interaction of DNMT3A with its partner TCL1A, in agreement with the MutPred2 score. See Supplementary Table 11 for predicted molecular mechanisms description for these mutations. Both disease mutations are predicted to affect secondary structure elements along with intrinsic disorder in the case of DNMT3A.

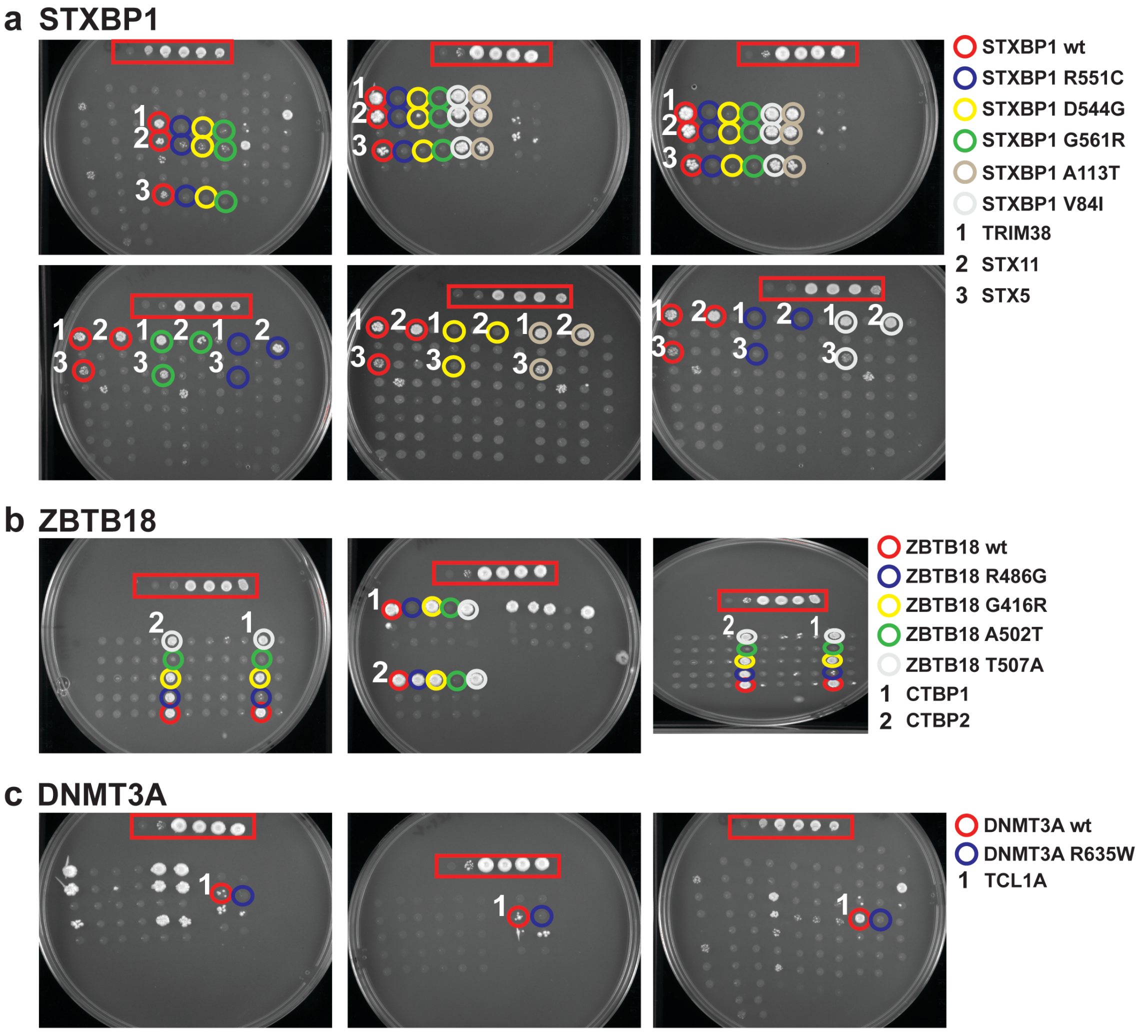

Supplementary Figure 9 | Full plate images for the experimental validation of the impact of mutations on protein-protein interactions using the yeast two-hybrid (Y2H) system. **(a)** Images of Sc-LT+3AT Y2H plate replicates for STXBP1 gene. **(b)** Images of Sc-LT+3AT Y2H plate replicates for ZBTB18 gene. (**c)** Images of Sc-LT+3AT Y2H plate replicates for DNMT3A gene. Scored interactions for all genes are marked with colored circles as per the legend. Interaction partners tested are marked with numbers. Auto-activation controls in each plate are marked with red square and are as follows (left to right); 1. pDEST-AD + pDEST-DB, 2. pDEST-AD-E2F1 + pDEST-DB-*CYH2*-pRB, 3. pDEST-AD-Jun + pDEST-DB-Fos, 4. pDEST-AD + pDEST-DB-Gal4, 5. pDEST-AD-dE2F1 + pDEST-DB-dDP, and 6. pDEST-AD-*CYH2*-dE2F1 + pDEST-DB-dDP (for details see Venkatesan *et al.*[58])

5 Supplementary Data

Supplementary Data 1 | Excel file containing a list of high-scoring amino acid variants with MutPred2 scores and predicted molecular mechanisms in neurodevelopmental cases vs. controls. Only those variants with general MutPred2 scores ≥ 0.67 (threshold corresponding to a 10% FPR) are shown. For each variant, the top three properties predicted to be affected are shown (ranked by posterior probability).

Supplementary Data 2 | Excel file containing list of all 1,345 features input to the MutPred2 model. Window lengths represent the number of positions considered around the substitution position. For example, a window length of 11 means all positions -5 and +5 of the substitution were considered when calculating the value for the give feature. NA for this column indicates that window lengths were not applicable in the computation of these features. For more details on how each feature was computed and/or encoded, see Supplementary Methods.

Supplementary References

1. Naderi-Manesh, H., Sadeghi, M., Arab, S. & Moosavi Movahedi, A.A. Prediction of protein surface accessibility with information theory. *Proteins* **42**, 452-459 (2001).
2. Kall, L., Krogh, A. & Sonnhammer, E.L. A combined transmembrane topology and signal peptide prediction method. *J. Mol. Biol.* **338**, 1027-1036 (2004).
3. Delorenzi, M. & Speed, T. An HMM model for coiled-coil domains and a comparison with PSSM-based predictions. *Bioinformatics* **18**, 617-625 (2002).
4. Xin, F. Methods for predicting functional residues in protein structures and understanding molecular mechanisms of disease. Computer Science and Informatics, Bloomington, IN (2012).
5. Lewis, B.A. *et al.* PRIDB: a protein-RNA interface database. *Nucleic Acids Res.* **39**, D277-282 (2011).
6. Agius, R. *et al.* Characterizing changes in the rate of protein-protein dissociation upon interface mutation using hotspot energy and organization. *PLoS Comput. Biol.* **9**, e1003216 (2013).
7. Disfani, F.M. *et al.* MoRFpred, a computational tool for sequence-based prediction and characterization of short disorder-to-order transitioning binding regions in proteins. *Bioinformatics* **28**, i75-83 (2012).
8. Huang, Z. *et al.* ASD v2.0: updated content and novel features focusing on allosteric regulation. *Nucleic Acids Res.* **42**, D510-516 (2014).
9. The UniProt Consortium. Uniprot: A hub for protein information. *Nucleic Acids Res.* **43**, D204-212 (2015).
10. Daróczy, Z. Generalized information functions. *Inform. Control* **16**, 36-51 (1970).
11. Uversky, V.N., Gillespie, J.R. & Fink, A.L. Why are “natively unfolded” proteins unstructured under physiologic conditions? *Proteins* **41**, 415-427 (2000).
12. Vihinen, M., Torkkila, E. & Riikonen, P. Accuracy of protein flexibility predictions. *Proteins* **19**, 141-149 (1994).
13. Eisenberg, D., Weiss, R.M. & Terwilliger, T.C. The hydrophobic moment detects periodicity in protein hydrophobicity. *Proc. Natl. Acad. Sci. USA* **81**, 140-144 (1984).
14. Goldsack, D.E. & Chalifoux, R.C. Contribution of the free energy of mixing of hydrophobic side chains to the stability of the tertiary structure of proteins. *J. Theor. Biol.* **39**, 645-651 (1973).
15. Kyte, J. & Doolittle, R.F. A simple method for displaying the hydropathic character of a protein. *J. Mol. Biol.* **157**, 105-132 (1982).
16. Vucetic, S., Brown, C.J., Dunker, A.K. & Obradovic, Z. Flavors of protein disorder. *Proteins* **52**, 573-584 (2003).
17. Peng, K. *et al.* Length-dependent prediction of protein intrinsic disorder. *BMC Bioinformatics* **7**, 208 (2006).

18. Radivojac, P. *et al.* Protein flexibility and intrinsic disorder. *Protein Sci.* **13**, 71-80 (2004).
19. Altschul, S.F. *et al.* Gapped BLAST and PSI-BLAST: a new generation of protein database search programs. *Nucleic Acids Res.* **25**, 3389-3402 (1997).
20. Riedmiller, M. & Braun, H. in IEEE International Conference on Neural Networks. 586-591 (IEEE, 1993).
21. Lupas, A., Van Dyke, M. & Stock, J. Predicting coiled coils from protein sequences. *Science* **252**, 1162-1164 (1991).
22. Baum, L.E., Petrie, T., Soules, G. & Weiss, N. A maximization technique occurring in the statistical analysis of probabilistic functions of markov chains. *Ann. Math. Stat.* **41**, 164-171 (1970).
23. Kabsch, W. & Sander, C. Dictionary of protein secondary structure: Pattern recognition of hydrogen-bonded and geometrical features. *Biopolymers* **22**, 2577-2637 (1983).
24. Rose, G.D. *et al.* Hydrophobicity of amino acid residues in globular proteins. *Science* **229**, 834-838 (1985).
25. Sander, C., Scharf, M. & Schneider, R. in Protein engineering. (eds. A.R. Rees, M.J.E. Sternberg & R. Wetzel) 89-115 (Oxford: IRL Press, 1992).
26. Cheng, J., Randall, A. & Baldi, P. Prediction of protein stability changes for single-site mutations using support vector machines. *Proteins* **62**, 1125-1132 (2006).
27. Karolchik, D. *et al.* The UCSC genome browser database: 2014 update. *Nucleic Acids Res.* **42**, D764-770 (2014).
28. Henikoff, S. & Henikoff, J.G. Amino acid substitution matrices from protein blocks. *Proc. Natl. Acad. Sci. USA* **89**, 10915-10919 (1992).
29. Dayhoff, M.O., Schwartz, R.M. & Orcutt, B.C. in Atlas of protein sequence and structure, Vol. 5 (3) 345-352 (1978).
30. Grantham, R. Amino acid difference formula to help explain protein evolution. *Science* **185**, 862-864 (1974).
31. Bromberg, Y. & Rost, B. SNAP: predict effect of non-synonymous polymorphisms on function. *Nucleic Acids Res.* **35**, 3823-3835 (2007).
32. Li, B. *et al.* Automated inference of molecular mechanisms of disease from amino acid substitutions. *Bioinformatics* **25**, 2744-2750 (2009).
33. Pei, J. & Grishin, N.V. Al2CO: calculation of positional conservation in a protein sequence alignment. *Bioinformatics* **17**, 700-712 (2001).
34. Kumar, S. *et al.* Evolutionary diagnosis method for variants in personal exomes. *Nat. Methods* **9**, 855-856 (2012).
35. Hsiao, T.L. & Vitkup, D. Role of duplicate genes in robustness against deleterious human mutations. *PLoS Genet.* **4**, e1000014 (2008).
36. Needleman, S.B. & Wunsch, C.D. A general method applicable to the search for similarities in the amino acid sequence of two proteins. *J. Mol. Biol.* **48**, 443-453 (1970).
37. Radivojac, P. *et al.* Calmodulin signaling: analysis and prediction of a disorder-dependent molecular recognition. *Proteins* **63**, 398-410 (2006).

38. Pejaver, V. *et al.* The structural and functional signatures of proteins that undergo multiple events of post-translational modification. *Protein Sci.* **23**, 1077-1093 (2014).
39. Sigrist, C.J. *et al.* New and continuing developments at prosite. *Nucleic Acids Res.* **41**, D344-347 (2013).
40. Dinkel, H. *et al.* The eukaryotic linear motif resource elm: 10 years and counting. *Nucleic Acids Res.* **42**, D259-266 (2014).
41. Calabrese, R. *et al.* Functional annotations improve the predictive score of human disease-related mutations in proteins. *Hum. Mutat.* **30**, 1237-1244 (2009).
42. Li, W. & Godzik, A. Cd-hit: a fast program for clustering and comparing large sets of protein or nucleotide sequences. *Bioinformatics* **22**, 1658-1659 (2006).
43. Liu, X., Wu, C., Li, C. & Boerwinkle, E. DBNSFP v3.0: a one-stop database of functional predictions and annotations for human nonsynonymous and splice-site snvs. *Hum. Mutat.* **37**, 235-241 (2016).
44. Potapov, V., Cohen, M. & Schreiber, G. Assessing computational methods for predicting protein stability upon mutation: good on average but not in the details. *Protein Eng. Des. Sel.* **22**, 553-560 (2009).
45. Khan, S. & Vihinen, M. Performance of protein stability predictors. *Hum. Mutat.* **31**, 675-684 (2010).
46. Sasidharan Nair, P. & Vihinen, M. Varibench: a benchmark database for variations. *Hum. Mutat.* **34**, 42-49 (2013).
47. Jain, S., White, M. & Radivojac, P. in Advances in Neural Information Processing Systems, NIPS 2016. 2693-2701 (Barcelona, Spain; December 2016).
48. Jain, S., White, M., Trosset, M.W. & Radivojac, P. Nonparametric semi-supervised learning of class proportions. *arXiv preprint arXiv:1601.01944* (2016).
49. Latinne, P., Saerens, M. & Decaestecker, C. in Proceedings of the Eighteenth International Conference on Machine Learning 298-305 (2001).
50. Saerens, M., Latinne, P. & Decaestecker, C. Adjusting the outputs of a classifier to new a priori probabilities: a simple procedure. *Neural Comput.* **14**, 21-41 (2002).
51. Jiang, Y. *et al.* An expanded evaluation of protein function prediction methods shows an improvement in accuracy. *Genome Biol.* **17**, 184 (2016).
52. Moult, J. *et al.* Critical assessment of methods of protein structure prediction (CASP)-round XII. *Proteins* **86 Suppl 1**, 7-15 (2018).
53. Karczewski, K.J. *et al.* Variation across 141,456 human exomes and genomes reveals the spectrum of loss-of-function intolerance across human protein-coding genes. *bioRxiv*, 531210 (2019).
54. Mort, M. *et al.* In silico functional profiling of human disease-associated and polymorphic amino acid substitutions. *Hum. Mutat.* **31**, 335-346 (2010).

55. Shoulders, M.D. & Raines, R.T. Collagen structure and stability. *Annu. Rev. Biochem.* **78**, 929-958 (2009).
56. Rauch, A. *et al.* Range of genetic mutations associated with severe non-syndromic sporadic intellectual disability: An exome sequencing study. *Lancet* **380**, 1674-1682 (2012).
57. Jiang, Y.H. *et al.* Detection of clinically relevant genetic variants in autism spectrum disorder by whole-genome sequencing. *Am. J. Hum. Genet.* **93**, 249-263 (2013).
58. Venkatesan, K. *et al.* An empirical framework for binary interactome mapping. *Nat. Methods* **6**, 83-90 (2009).
