# Supplementary material for: Inferring the molecular and phenotypic impact of amino acid variants with MutPred2
Source: Nat Commun. 2020 Nov 20;11:5918. doi: 10.1038/s41467-020-19669-x (PMC7680112; doi:10.1038/s41467-020-19669-x)
Supplement: Supplementary file 2 — Descriptions of Additional Supplementary Files [file 41467_2020_19669_MOESM2_ESM.pdf]

Descriptions of Additional Supplementary Files

Supplementary Data 1

Description: A list of high-scoring amino acid variants with MutPred2 scores and predicted molecular mechanisms in neurodevelopmental cases vs. controls. Only those variants with general MutPred2 scores ≥ 0.67 (threshold corresponding to a 10% FPR) are shown. For each variant, the top three properties predicted to be affected are shown (ranked by posterior probability).

Supplementary Data 2

Description: A list of all 1,345 features input to the MutPred2 model. Window lengths represent the number of positions considered around the substitution position. For example, a window length of 11 means all positions -5 and +5 of the substitution were considered when calculating the value for the give feature. NA for this column indicates that window lengths were not applicable in the computation of these features. For more details on how each feature was computed and/or encoded, see Supplementary Methods.
