# Supplementary material for: Inferring the molecular and phenotypic impact of amino acid variants with MutPred2
Source: Nat Commun. 2020 Nov 20;11:5918. doi: 10.1038/s41467-020-19669-x (PMC7680112; doi:10.1038/s41467-020-19669-x)
Supplement: Supplementary file 5 — Reporting Summary [file 41467_2020_19669_MOESM5_ESM.pdf]

nature research

Corresponding author(s): Predrag Radivojac

Last updated by author(s): Aug 31, 2020

Reporting Summary

Nature Research wishes to improve the reproducibility of the work that we publish. This form provides structure for consistency and transparency in reporting. For further information on Nature Research policies, see our Editorial Policies and the Editorial Policy Checklist.

Statistics

For all statistical analyses, confirm that the following items are present in the figure legend, table legend, main text, or Methods section.

n/a	Confirmed	
☐	☒	The exact sample size (n) for each experimental group/condition, given as a discrete number and unit of measurement
☒	☐	A statement on whether measurements were taken from distinct samples or whether the same sample was measured repeatedly
☐	☒	The statistical test(s) used AND whether they are one- or two-sided<br>*Only common tests should be described solely by name; describe more complex techniques in the Methods section.*
☒	☐	A description of all covariates tested
☐	☒	A description of any assumptions or corrections, such as tests of normality and adjustment for multiple comparisons
☐	☒	A full description of the statistical parameters including central tendency (e.g. means) or other basic estimates (e.g. regression coefficient) AND variation (e.g. standard deviation) or associated estimates of uncertainty (e.g. confidence intervals)
☐	☒	For null hypothesis testing, the test statistic (e.g. F, t, r) with confidence intervals, effect sizes, degrees of freedom and P value noted<br>*Give P values as exact values whenever suitable.*
☒	☐	For Bayesian analysis, information on the choice of priors and Markov chain Monte Carlo settings
☒	☐	For hierarchical and complex designs, identification of the appropriate level for tests and full reporting of outcomes
☒	☐	Estimates of effect sizes (e.g. Cohen's d, Pearson's r), indicating how they were calculated

Our web collection on statistics for biologists contains articles on many of the points above.

Software and code

Policy information about availability of computer code

Data collection	All training data was collected from publicly available sources such as the Human Gene Mutation Database, UniProt, dbSNP and the UCSC Genome Browser. Custom code was written to extract these data and present them in the file hosted at http://mutpred.mutdb.org
Data analysis	MutPred2 was written in MATLAB with only one external dependency (a legacy version of PSI-BLAST). We have made the source code available on Github and also provide access to a compiled executable and web-server through the MutPred URL above. Details are provided in the "Code Availability" statement.

For manuscripts utilizing custom algorithms or software that are central to the research but not yet described in published literature, software must be made available to editors and reviewers. We strongly encourage code deposition in a community repository (e.g. GitHub). See the Nature Research guidelines for submitting code & software for further information.

Data

Policy information about availability of data

All manuscripts must include a data availability statement. This statement should provide the following information, where applicable:

- Accession codes, unique identifiers, or web links for publicly available datasets
- A list of figures that have associated raw data
- A description of any restrictions on data availability

A modified version of the training data for MutPred2 that does not contain variants exclusively from HGMD is available at http://mutpred.mutdb.org/#dload. Since we used the professional version of HGMD, restrictions apply to the availability of these data. These variants are however available under the appropriate license agreement at http://www.hgmd.cf.ac.uk/ac/index.php.

Field-specific reporting

Please select the one below that is the best fit for your research. If you are not sure, read the appropriate sections before making your selection.

☒ Life sciences ☐ Behavioural & social sciences ☐ Ecological, evolutionary & environmental sciences

For a reference copy of the document with all sections, see nature.com/documents/nr-reporting-summary-flat.pdf

Life sciences study design

All studies must disclose on these points even when the disclosure is negative.

Sample size	This study primarily focuses on methodology development and hypothesis generation. However, it was ensured that sample sizes were sufficient to train machine learning models and leveraged data from existing resources and databases to the best extent possible.
Data exclusions	Since the data in this study were primarily used for methodology development, they were restricted to what was available in publicly available variant databases and resources. Subsequent curation and exclusion criteria have been described in the Methods section and Supplementary Materials as exhaustively as possible.
Replication	This study primarily focuses on methodology development and hypothesis generation. Experimental work and results were restricted to the yeast two-hybrid (Y2H) assays performed as validation of predictions. All Y2H screens were repeated three times in independent experiments and only interactions that scored as positives at least twice were considered as positives.
Randomization	This study primarily focuses on methodology development and hypothesis generation, and hence randomization was not relevant.
Blinding	Blinding was not relevant to the study. Implicit blinding was achieved through cross-validation and holdout independent set evaluations.

Reporting for specific materials, systems and methods

We require information from authors about some types of materials, experimental systems and methods used in many studies. Here, indicate whether each material, system or method listed is relevant to your study. If you are not sure if a list item applies to your research, read the appropriate section before selecting a response.

Materials & experimental systems

n/a	Involved in the study
☒	☐ Antibodies
☒	☐ Eukaryotic cell lines
☒	☐ Palaeontology and archaeology
☒	☐ Animals and other organisms
☒	☐ Human research participants
☒	☐ Clinical data
☒	☐ Dual use research of concern

Methods

n/a	Involved in the study
☒	☐ ChIP-seq
☒	☐ Flow cytometry
☒	☐ MRI-based neuroimaging
